# Scaling relationships and theory for vibrational frequencies of adsorbates on transition metal surfaces

**DOI:** 10.1038/s41467-017-01983-6

**Published:** 2017-11-29

**Authors:** Joshua L. Lansford, Alexander V. Mironenko, Dionisios G. Vlachos

**Affiliations:** 1 Department of Chemical & Biomolecular Engineering, University of Delaware, Newark, DE 19716 USA; 2 Catalysis Center for Energy Innovation, University of Delaware, 221 Academy Street, 250R, Newark, DE 19716 USA

## Abstract

Adsorbate vibrational excitations are an important fingerprint of molecule/surface interactions, affecting temperature contributions to the free energy and impacting reaction rate and equilibrium constants. Furthermore, vibrational spectra aid in identifying species and adsorption sites present in experimental studies. Despite their importance, knowledge of how adsorbate frequencies scale across materials is lacking. Here, by combining previously reported experimental data and our own density-functional theory calculations, we reveal linear correlations between vibrational frequencies of adsorbates on transition metal surfaces. Through effective-medium theory, linear muffin-tin orbital theory, and the *d*-band model, we rationalize the squares of the frequencies to be fundamentally linear in their scaling across transition metal surfaces. We identify the adsorbate-binding energy as a descriptor for certain molecular vibrations and rigorously relate errors in frequencies to errors in adsorption energies. We also discuss the impact of scaling on surface thermochemistry and adsorbate coverage.

## Abstract (web summary)

Vibrational excitations are a fingerprint of molecule–surface interactions, but knowing how they scale across materials is tricky. Here, the authors discover correlations between the vibrational frequencies of adsorbates on transition metals, developing a predictive theory to allow interpretation of complex experimental spectra.

## Introduction

A breakthrough in computational catalysis was the introduction of linear scaling relations (LSRs), by Nørskov and co-workers^1^, which link the binding energy of a partially hydrogenated adsorbate AH_*X*_ $\left( {{\mathrm{\Delta }}E^{{\mathrm{AH}}_X}} \right)$ to the binding energy of its respective atomic adsorbate A (Δ*E* ^A^) across transition metal surfaces,

1 $${\mathrm{\Delta }}E^{{\mathrm{AH}}_X} = m_E{\mathrm{\Delta }}E^{\mathrm{A}} + b_E,$$

where the slope, *m*_*E*_, depends on the valencies of A and AH_*X*_. LSRs provide a means to estimate thermodynamic properties of reaction intermediates on heterogeneous catalyst surfaces, enabling in silico catalyst screening^2, 3^. While LSRs can predict the scaling of binding energies, there is not yet a way to scale zero-point energies (ZPE) and temperature contributions to Gibbs free energy across surfaces. These temperature effects, which are composed of a molecule’s heat capacity and entropy, result entirely from vibrations for immobilized chemisorbed surface species^4^. As vibrations of adsorbates are often considered to be invariant with respect to the metal surface^5^, temperature effects and pre-exponentials are typically taken to be constant in microkinetic modeling of heterogeneously catalyzed systems^6, 7^.

There is evidence suggesting that frequencies should scale with electronic energy of the relevant chemical bonds. For example, both experimental gas-phase diatomic frequencies, and density functional theory (DFT)-determined force constants scale with bond dissociation energy^8, 9^. A DFT study of ammonia adsorbed on transition metals indicates that N–H vibrational stretching and bending frequencies experience slight trends with binding energy^10^. Furthermore, the C–O stretching vibration for chemisorbed carbon monoxide varies linearly with coverage below 300 K^11^. Coverage effects on frequencies can be separated into static and dynamic frequency shifts^12–15^. The former can be treated as a consequence of the metal’s *d*-band centre shift with coverage, similar to the effects on adsorption energy^16^, implying that frequencies should relate to energies, but the explicit dependence is lacking^17^. On a more fundamental level, the vibrational force constants can be linked analytically to the electron density^18,^ and DFT is based on the knowledge that the electronic density completely specifies a systems energy^19^.

Here we develop vibrational scaling relations (VSRs) for CH_*X*_, NH_*X*_, and OH_*X*_ species, as well as for CH_2_CH_3_, adsorbed on transition metal surfaces. Using effective-medium theory (EMT)^20–24^ and linear muffin-tin orbital (LMTO)^21, 25, 26^ theory, we derive an expression that fundamentally relates the slopes of the LSRs and the VSRs. On the basis of this theory, we successfully predict the VSRs from the corresponding LSRs, adsorbate geometry, and reduced mass on any single transition metal surface. We show the influence of the VSRs and vibrational frequencies on thermodynamic and kinetic model predictions and rationalize their accuracy.

## Results

### Vibrational scaling theory and connection to energy scaling

Here we provide the essential theory and equations for understanding vibrational scaling. A more detailed derivation with discussion is provided in Supplementary Note 1. According to tight-binding theory and the *d*-band model^27^, the adsorption energy (Δ*E*_ads_) can be separated into *sp*-band (Δ*E*_*sp*_) and *d*-band (Δ*E*_*d*_) contributions:

2 $${\mathrm{\Delta }}E = {\mathrm{\Delta }}E_{sp} + {\mathrm{\Delta }}E_d.$$

Furthermore, every bulk transition metal has its outer *s*-orbital half-filled, and the coupling of the adsorbate to the metal *sp*-band is constant across transition metals^27^. The slope of the LSR in Eq. (1) is then attributed to the coupling of the adsorbate’s *s* and *p* orbitals with the transition metal’s *d*-band^1^. Consequently, we can reformulate Eq. (1) as follows:

3 $${\mathrm{\Delta }}E_d^{{\mathrm{AH}}_X} = m_E{\mathrm{\Delta }}E_d^{\mathrm{A}}.$$

Within the harmonic approximation, the frequency (*v*) of a normal mode is given according to

4 $$\nu = \frac{1}{{2\pi }}\sqrt {\frac{k}{\mu }} ,$$

with reduced mass (*μ*) and force constant (*k*), where *k* is the second derivative of the potential energy surface (PES) at equilibrium with respect to the direction of a normal mode displacement.

Due to the additive nature of adsorption energy according to Eq. (2), *k* can be taken as a sum of contributions from adsorbate coupling to the metal *sp*-band (*k*_*sp*_) and *d*-band (*k*_*d*_):

5 $$k = k_{sp} + k_d.$$

From EMT, Δ*E*_*sp*_ is exponential with respect to separation between the adsorbate and metal surface. We show in Supplementary Fig. 1 that a Morse potential adequately describes this interaction. The second derivative of any sum of exponential functions results in *k*_*sp*_ being proportional to an adsorbate-dependent constant *(η*) and the *sp* contribution to the adsorbate-binding energy at equilibrium (Δ*E*_*sp*_).

According to Anderson’s LMTO theory, the *d*-band contribution to binding energy (Δ*E*_*d*_) is proportional to adsorbate- and metal-dependent constants *M*_A_ and *M*_M_, and is related to the distance (*r*) between the nuclei of the adsorbate and the nearest metal atoms. Formulating Anderson’s expression to account for the existence of both adsorbate *s* and *p* states results in the following:

6 $${\mathrm{\Delta }}E_d \propto \left( {M_{\mathrm{A}}M_{\mathrm{M}}} \right)^2\left( {\frac{{f_s}}{{r^{n_s}}} + \frac{{f_p}}{{r^{n_p}}}} \right).$$

Here, *f*_*s*_ and *f*_*p*_ represent the fractional contributions of the adsorbate’s *s* and *p* orbitals to the adsorbate-metal bond, respectively, such that *f*_*s*_ + *f*_*p*_ = 1 and

7 $$n_a = 2\left( {l_{\mathrm{a}} + l_d + 1} \right)$$

Constants *n*_*s*_ and *n*_*p*_ depend on the angular momenta of the adsorbate (*l*_a_) and metal *d* orbitals (*l*_*d*_)^21, 25^. Inserting second derivatives of Δ*E*_*sp*_ and Δ*E*_*d*_ into Eq. (4) allows Eq. (8) to describe any frequency driven by the adsorbate-metal interaction:

8 $$\nu = \frac{1}{{g\left( \theta \right)2\pi \sqrt \mu }}\sqrt {\eta {\mathrm{\Delta }}E_{sp} + R_n{\mathrm{\Delta }}E_d}$$

where *μ* is the reduced mass of the corresponding normal mode, *g*(*θ*) relates the displacement of the normal mode to a change in *r*, and *R*_*n*_ is expressed in terms of previously defined quantities:

9 $$R_n = \frac{{f_sn_s\left( {n_s + 1} \right)r^{n_p} + f_pn_p\left( {n_p + 1} \right)r^{n_s}}}{{r^2\left( {f_sr^{n_p} + f_pr^{n_s}} \right)}}.$$

We note that *η* is negative and *R*_*n*_ is positive. The importance of their signs will be discussed later.

In this work, *f*_*s*_ and *f*_*p*_ were estimated from the total number of adsorbate’s *s* and *p* electrons in the atom bound to the metal. Thus, for all OH_*X*_, NH_*X*_, and CH_*X*_ species, *f*_*s*_ was equal to 1/3, 2/5, and 1/2, respectively, resulting in the following equality:

10 $$f_s^{\mathrm{A}} = f_s^{{\mathrm{AH}}_X}$$

A projected density of states analysis suggests that 2/5 may be a more appropriate value for CH_*X*_ species. We also find that the value of *f*_*s*_ does not affect the predicted scaling significantly when Eq. (10) holds (Supplementary Fig. 2). Here we shall address the case where *g*(*α*) = 1, which is strictly true only for the perpendicular frequency (*ν*_⊥_) at an atop site.

By representing *ν* ^AH^_X_ with Eq. (8), we can find its scaling with respect to *ν* ^A^. First, because ${\mathrm{\Delta }}E_d^{{\mathrm{AH}}_X}$ is described according to Eq. (3), we can replace ${\mathrm{\Delta }}E_d^{{\mathrm{AH}}_X}$ in 8 with its functional dependence on *ν* ^A^. Then a series of substitutions and taking the derivative of *ν* ^AH^_X_ with respect to *ν* ^A^ results in

11 $$\frac{{d\nu ^{\mathrm{AH}_X}}}{{{\mathrm{d}}\nu ^{\mathrm{A}}}} = \sqrt {\frac{{\mu ^{\mathrm{A}}}}{{\mu ^{{\mathrm{AH}}_X}}}} \frac{{R_n^{{\mathrm{AH}}_X}m_E\sqrt {\eta ^{\mathrm{A}}{\mathrm{\Delta }}E_{sp}^{\mathrm{A}} + {\mathrm{\Delta }}E_d^{\mathrm{A}}R_n^{\mathrm{A}}} }}{{R_n^{\mathrm{A}}\sqrt {\eta ^{{\mathrm{AH}}_X}{\mathrm{\Delta }}E_{sp}^{{\mathrm{AH}}_X} + m_E{\mathrm{\Delta }}E_d^{\mathrm{A}}R_n^{{\mathrm{AH}}_X}} }},$$

where *m*_*E*_ is the LSR slope, *μ* is assumed to be the total mass of the adsorbate, and *R*_*n*_ is determined from Eq. (9). For AH_*X*_ species, the light mass of hydrogen allows the reduced mass of the species to be approximated as the total mass.

Upon substitution of Eq. (8) into Eq. (11) and subsequent simplifications, as outlined in Supplementary Note 1, we get the expression for the first derivative of (*ν* ^AH^_X_)^2^ with respect to (*ν* ^A^)^2^:

12 $$\frac{{{\mathrm{d}}\left( {\nu ^{{\mathrm{AH}}_X}} \right)^2}}{{{\mathrm{d}}\left( {\nu ^{\mathrm{A}}} \right)^2}} = m_E\frac{{\mu ^{\mathrm{A}}}}{{\mu ^{{\mathrm{AH}}_X}}}\left( {\frac{{R_n^{{\mathrm{AH}}_X}}}{{R_n^{\mathrm{A}}}}} \right)$$

where the terms in *R*_*n*_ can be determined from the equilibrium geometry and electronic structure of an adsorbate on a reference metal. In this work, the reference metal is taken to be Pt. Upon integration, we obtain a linear scaling relationship between the squared frequencies of AH_*X*_ and A:

13 $$\left( {\nu^{{\mathrm{AH}}_X}} \right)^2 = m_\nu\left( {\nu^{\mathrm{A}}} \right)^2 + b_v$$

with slope (*m*_*v*_) given by Eq. (12), such that $m_\nu = \frac{{{\mathrm{d}}\left( \nu ^{{\mathrm{AH}}_X} \right)^2}}{{{\mathrm{d}}\left( {\nu ^{\mathrm{A}}} \right)^2}}$. The intercept (*b*_*v*_) is derived in Supplementary Note 2 by setting *k*_*d*_ = −*k*_*sp*_ and, like the LSR intercept^1^, is independent of the *d*-band contribution to binding energy:

14 $$  {b_\nu} = \frac{ {\sqrt {{\eta ^{{\mathrm{AH}}_ X}}\Delta {E_{sp}^{{\mathrm{AH}}_X}} - {\frac{ {R_n^{{\mathrm{AH}}_X}}} {{R_n^{\mathrm{A}}}}} {m_E}{\eta ^{\mathrm{A}}} \Delta {E_{sp}^{\mathrm{A}}} }}}     {2\pi \sqrt {\mu ^{{\mathrm{AH}}_X}} }.$$

The slope and intercept are specific to any A/AH_*X*_ pair of adsorbates. We call the scaling relations of Eq. (13) vibrational scaling relationships (VSRs).

These VSRs should hold for any vibrational mode primarily driven by interaction of the adsorbate with the metal. We test the predictive power of the derived relations in the following section by calculating DFT normal mode frequencies for AH_*X*_ adsorbates and investigating trends across transition metals.

### Scaling of normal mode vibrations calculated using DFT

**Fig. 1:**
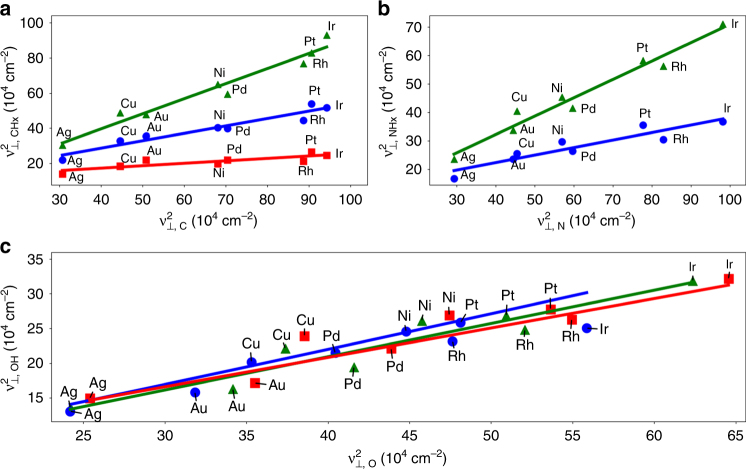
Scaling of the *ν*_⊥_ modes for AH_*X*_ adsorbates with their atomic counterparts from DFT. Species are **a** CH_*X*_, **b** NH_*X*_, and **c** OH_*X*_. Adsorption is on the atop site of (100) transition metal surfaces. For **a** and **b**, respectively: green triangles = CH and NH, blue circles = CH_2_ and NH_2_, and red squares = CH_3_. For OH_*X*_ species **c**, *ν*_⊥_ frequencies on the (110) and (111) facets are also included and green triangles = (100), blue circles = (111) and red squares = (100). The slopes of the regressed lines in **a** are 0.14, 0.42, and 0.86 for the red, blue, and green lines, respectively. In **b**, the slopes of the regressed lines are 0.26 and 0.64 for the blue and green lines, respectively. In **c** the slopes of the regressed lines are 0.43, 0.42, and 0.48 for the red, blue and green lines, respectively. All intercepts are positive

In Fig. 1, we report DFT-computed frequencies of adsorbed species based on the harmonic approximation with normal mode analysis. The data support the existence of linear correlations between squared vibrational frequencies of AH_*X*_ and atomic A species across transition metal surfaces, for A=C, N, and O, and *X* = 1 up to one less than the valency of the atomic species. Due to the relatively narrow range of frequencies, the error in predicting frequencies is not significantly higher when a linear model is used. The average root mean square error in predicting frequencies of these models is 22 ± 5 cm^−1^ (square model) and 23 ± 5 cm^−1^ (linear model), at the atop site for all facets. The standard errors in the regressed VSR slopes for O/OH, NH/NH_2_, and CH_2_/CH_3_, all with valency ratios of 1/2, are 0.062, 0.047, and 0.051, respectively, on the (100) surface when the scaling is with respect to frequency squared and 0.089, 0.055, and 0.067, respectively, for a linear fit. While a linear model fits the data reasonably well, the squared relation arises naturally from theory and, as discussed later, can be used to predict the VSR slopes over a wide range of values.

Comparison of ν_⊥_ (Fig. 1) and ν_||_ (Supplementary Note 3 and Supplementary Figs. 3–5) AH_*X*_ frequency trends reveal universal linear scaling behaviour, as frequencies of species adsorbed on top sites, three and four-fold hollow sites, and bridge sites all correlate. Interestingly, the VSR intercepts are positive, as opposed to negative for their respective LSRs. A negative intercept for the LSR is a result of its slope being less than one, as the adsorption energy on pure transition metals is more negative for atomic adsorbates than their hydrogenated counterparts. The positive intercept for the VSR is then expected from Eq. (14), as *m*_*E*_ < 1 for all A/AH_*X*_ pairs, and $\frac{{R_n^{{\mathrm{AH}}_X}}}{{R_n^{\mathrm{A}}}}$ is usually less than one as well. As already mentioned, for the AH_*X*_ adsorbates considered here, the fraction of the adsorbate *s* and *p* orbitals interacting with the metal *d*-band was taken to be constant. Thus, *R*_*n*_ is primarily a function of the distance between the adsorbate and the metal atom. These distances are provided in Supplementary Table 1.

**Fig. 2:**
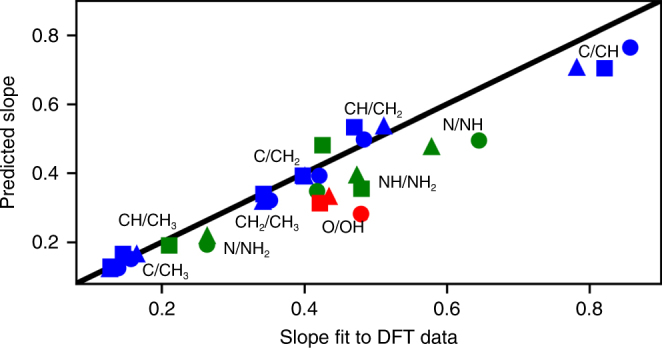
Predicted VSR slopes for AH_*X*_ species. Values are predicted based on Eq. (12). Labels indicate adsorbate A/B for CH_*X*_ (blue), NH_*X*_ (green), and OH_*X*_ (red). The black line indicates parity. The fitted slopes to DFT data are from the data in Fig. 1 and fit according to Eq. (13). Included are the perpendicular frequencies of the adsorbates on the atop sites of the (100) (circles), (110) (triangles), and (111) (squares) facets

In Fig. 2, the theoretical slopes of the VSRs predicted by Eq. (12) are compared to those found by fitting the DFT-calculated normal mode frequencies shown in Fig. 1, according to Eq. (13). When the LSR and VSR fittings exhibit small standard errors, such as for CH_*X*_ species, the slopes match well. To explain deviations in O/OH, we note that the value of $R_n^{{\mathrm{AH}_X}}$/$R_n^{\mathrm{A}}$ is roughly constant across metals, so that the uncertainty in the O/OH VSR is primarily associated with the variance in its LSR (Supplementary Table 1). For comparison, LSR slopes for O/OH, NH/NH_2_, and CH_2_/CH_3_, all with valency ratios of 1/2, have standard errors of 0.12, 0.04, and 0.05, respectively, on the (100) surface at the atop site. The standard errors for other NH_*X*_ and CH_*X*_ LSR slopes are about 0.05. Our model successfully predicts VSR slopes that range from 0.14 (C/CH_3_) to 0.86 (C/CH).

Our model is based on an assumption that *f*_*s*_ and *f*_*p*_ are invariant within the same class of AH_*X*_ species. While this is generally true^28^, a projected density of states analysis (Supplementary Table 2) onto atom A shows that the fraction of electron density attributed to *p*-electrons monotonically increases by a small amount with increasing *X* for all adsorbates. Such observations have already been reported for CH_*X*_ species on Pd^29^. If the *p* character of the adsorbate-bonding orbital increases in a similar manner, this would explain the tendency of our model to slightly under-predict the VSR slope between two species AH_*X*1_/AH_*X*2_ when *X*_2_ > *X*_1._

The results for frequency scaling in this work are valid for normal modes primarily driven by interaction of the adsorbate with the metal surface atoms, such as metal-adsorbate *ν*_⊥_ and *ν*_||_ stretches, as well as δ_⊥_ rotations. For molecular adsorbates, the *ν*_||_ mode is often dependent on surface geometry. See Supplementary Note 3 and Supplementary Figs. 3–5 for scaling of *ν*_||_ frequencies and the section on extension to complex molecules for δ_⊥_ rotations.

### A/B vibrational scaling

To this point, we have only covered scaling of A/AH_*X*_ species. Calle-Vellejo et al. showed that linear scaling holds for binding energies of AH_*X*_ and BH_*X*_, where A and B have the same number of valence electrons, such as for CH_*X*_ and SiH_*X*_ species^30^. They also showed that for the adsorption energy of O (Δ*E*_O_) and N (Δ*E*_N_) on bimetallic alloys of Pt, a general scaling relation exists:

15 $${\mathrm{\Delta }}E_{\mathrm{O}} = \gamma E_{\mathrm{N}} + g\left( {\omega _{T_1},\omega _{T_2}} \right).$$

**Fig. 3:**
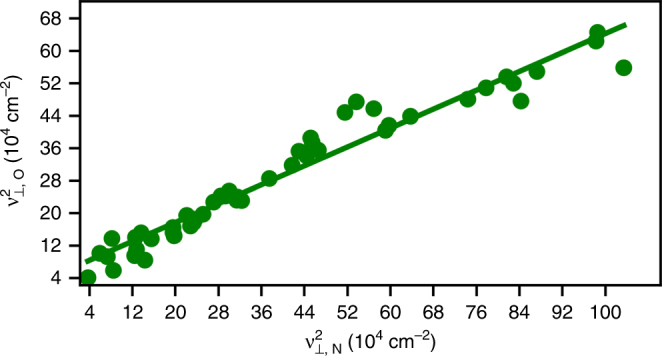
DFT *ν*_⊥_ normal mode frequencies for atomic O vs. N. The data include hollow, bridge, and atop site adsorption on the (111), (110), and (100) facets of Ag, Au, Cu, Ir, Pd, Pt, and Rh. The slope of the regressed line is 0.58 and the intercept is 59,800 cm^−2^

**Fig. 4:**
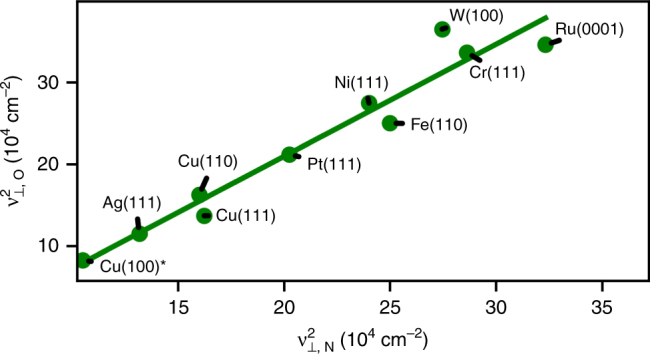
Experimental *ν*_⊥_ frequencies for atomic O vs. N. Label Cu(100)* indicates ordered adsorbate layers at high coverage. Atomic O adsorption is typically at low coverage. Atomic N was often co-adsorbed with other species. The slope of the regressed line is 1.37 and the intercept is −64,500 cm^−2^

In Eq. (15), *γ* is a constant with respect to *E*_N_, and *g* is a function of the number of valence electrons ($\omega _{T_1}$ and $\omega _{T_2}$) in the transition metals. It is straightforward to show that if $\frac{{\partial \gamma }}{{\partial r}}$ is small and $\frac{{\partial ^2g}}{{\partial r^2}}$ is constant, the squares of the frequencies should scale with a slope that is approximately equal to *γ*. The DFT data plotted in Fig. 3 and the experimental data in Fig. 4 highlight that frequencies between species A/B do indeed scale, and that this scaling is universal across various sites and facets, as all frequencies lie on the same line.

Figure 3 shows perpendicular frequency scaling of atomic N vs. atomic O on several sites and facets of fcc metals from DFT. The best fit value of *γ* used in scaling adsorption energy on Pt-bimetallic alloys was 0.67^30^. Our value of 0.58 for the VSR slope indicates that either $\frac{{\partial \gamma }}{{\partial r}}$ is not zero or $\frac{{\partial ^2g}}{{\partial r^2}}$ is not constant, but the minor deviation from 0.67 suggests such assumptions are a good approximation. Accounting for *g*(*θ*), as we do in Supplementary Note 1 for AH_*X*_ frequencies at hollow and bridge sites, results in a slope of 0.63.

In Fig. 4, we plot previously reported low-coverage experimental vibrational frequencies of atomic O, and N on ideal metal surfaces, obtained by High-Resolution electron Energy Loss Spectroscopy (HREEL) at temperatures between 30 and 500 K^31–49^. Figure 4 is not expected to match Fig. 3 for several reasons: (1) the infinite metal mass approximation in DFT calculations, (2) the possibility of different experimental binding sites for atomic O and N, and (3) presence of co-adsorbed species during experimental measurement of atomic N frequencies.

The discovered universality of vibrational scaling for chemically different adsorbates and metals evidently stems from the universal electronic structure description of bonding on transition metal surfaces^26, 50^. Therefore, we expect similar correlations should exist between vibrational frequencies of more complex species as well.

### Extension to more complex molecules

**Fig. 5:**
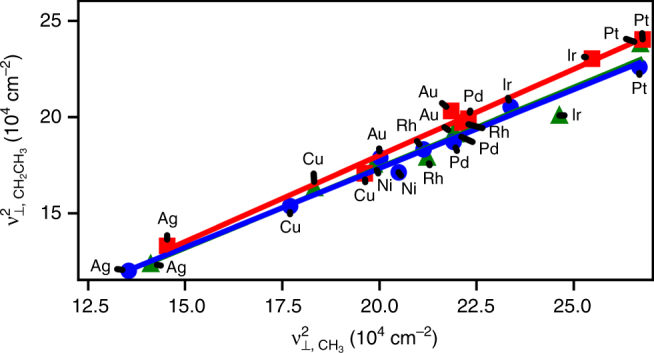
Scaling of *ν*_⊥_ for CH_2_CH_3_ vs. CH_3_. The data is calculated from DFT at the atop site. The data include vibrations on (100) (green triangles), (111) (blue circles), and (110) (red squares) transition metal surfaces. The slopes of the regressed lines are 0.90, 0.82, and 0.83 for the red, blue and green lines, respectively

To determine whether the VSR theory is applicable to large molecules, we calculated frequencies for CH_2_CH_3_ adsorbed on atop sites. Figure 5 indicates that ν_⊥_(Metal-CH_2_CH_3_) frequencies correlate with the corresponding frequencies of CH_3_ at the atop site. While all atoms in CH_2_CH_3_ are displaced in this normal mode, the frequency is primarily attributed to the stretching of the metal-carbon bond. Because the reduced mass can only be calculated exactly for a two-body system, the theoretical scaling relation for the AH_*X*_ species, Eq. (12), provides bounds. Generally, an adsorbate’s reduced mass for a *ν*_⊥_ mode will lie between the mass of the adsorbate atom nearest to the surface and the total mass of the adsorbed molecule. The predicted frequency slope for *ν*_⊥_ scaling of CH_2_CH_3_ vs. CH_3_ is between 0.57 and 1.10, when the reduced mass is set to the total mass of the adsorbate or the mass of the bonding carbon atom, respectively. The actual slope is between 0.82 and 0.90 depending on facet. Furthermore, while scaling on more complex surfaces is expected to exist^51^, geometric effects on the reduced mass that are surface dependent are likely to be important.

### Scaling of frequencies with adsorption energy

The correlation between frequencies and adsorption energies is fairly complex, due to the force constant involving contributions from both the metal *sp*-band and *d*-band (*η*Δ*E*_*sp*_ and Δ*E*_*d*_ *R*_*n*_, respectively) according to Eq. (8). As the magnitude of Δ*E*_*d*_ *R*_*n*_ increases relative to *η*Δ*E*_*sp*_, the scaling takes a square root dependence on energy. In contrast, when *η*Δ*E*_*sp*_ is dominant, the frequency becomes independent of adsorption energy. The order of the scaling therefore lies between 0 and 1/2. Substituting Eq. (2) into Eq. (8) results in the following frequency dependence on adsorption energy,

16 $$\nu^2 = \frac{1}{{4\pi^2 \mu }}\left( {\eta {\mathrm{\Delta }}E_{sp} + R_n\left( {{\mathrm{\Delta }}E_{{\mathrm{ads}}} - {\mathrm{\Delta }}E_{sp}} \right)} \right).$$

Because *R*_*n*_ is positive and approximately constant (see Supplementary Note 1), and frequencies increase with increasing binding energy, *η* must increase in magnitude as binding energy increases. Allowing *η* to be a linear function of Δ*E*_*d*_ gives the following empirical relation for the dependence of frequency on adsorption energy:

17 $$\nu ^2 = \alpha {\mathrm{\Delta }}E_{{\mathrm{ads}}} + \beta$$

where *α* and *β* are constants.

**Fig. 6:**
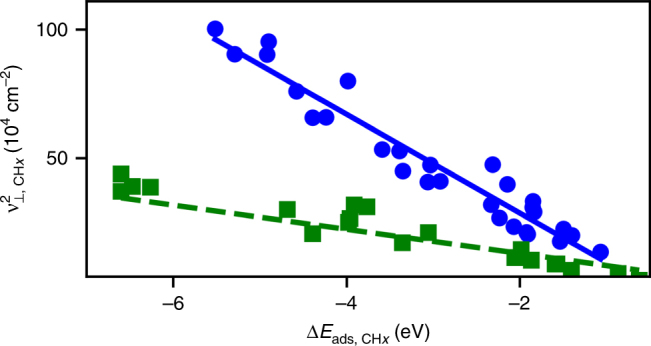
Scaling of frequencies with adsorption energy. Frequencies are for the *ν*_⊥_ mode and calculated from DFT for CH_*X*_ species, where *X* ≥ 0. Included are modes and adsorption energies at atop sites (blue circles) and fcc hollow sites (green squares). Fits to Eq. (17) are shown by solid blue and dashed green lines, respectively. The slopes of the regressed lines are −192,000 and −63,100 for the blue and green lines, respectively. The intercepts are also both negative

Figure 6 depicts scaling of *ν*_⊥_ DFT-calculated frequencies of CH_*X*_ species, for *X* = 0 to 3, on fcc hollow (green squares) and atop (blue circles) sites for close-packed surfaces of fcc metals against the electronic adsorption energies. Scaling of *ν*_⊥_ with adsorption energy for OH_*X*_ and NH_*X*_, as well as *ν*_||_ for CH_*X*_, NH_*X*_, and OH_*X*_, are shown in Supplementary Note 4 and Supplementary Figs. 6 and 7. The data reveal that (1) adsorbates with larger frequency and adsorption energy ranges have less variance, and (2) smaller equilibrium distances between the adsorbate and metal atoms, result in greater *d*-band contributions (Δ*E*_*d*_ *R*_*n*_) to frequency scaling. These characteristics are expected from Eqs. (8), (9) and (16). Frequencies, which can be measured spectroscopically, provide a means of estimating and comparing adsorption energies at different sites and on different surfaces.

### Interpreting complex experimental vibrational spectra

Based on selection rules and our own scaling studies of experimental O_2_ and O frequencies in the Supporting Information, we identify frequencies associated with molecular oxygen in the range of 500 and 800 cm^−1^ as frustrated rotations (*δ*_⊥_). Scaling frequencies provide additional information to single metal frequency studies. Conclusions drawn from previous experimental studies on a single metal are discussed in Supplementary Note 5. Because the *δ*_⊥_(M–O_2_) motion is primarily perpendicular to the surface, its corresponding HREELS peak should be much more intense than any ν(O–O) stretch where the intra-oxygen bond is parallel to the surface^52^. Partially because of these selection rules, we suggest frequencies identified experimentally between 600 and 1000 cm^−1^ are a combination of O–O stretches at the bridge site and metal-oxide stretches on stronger binding metals. The frequency of both of these experimentally observed modes increases as the atomic oxygen frequency increases, as seen in Supplementary Fig. 8, which is consistent with modes driven by interaction of the oxygen species with the surface, not a pure O–O stretch.

**Fig. 7:**
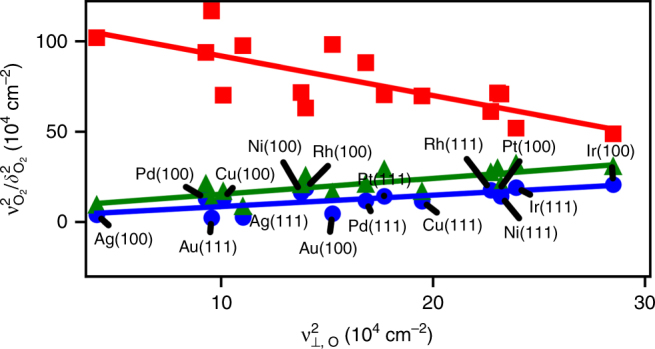
Scaling of O_2_ frequencies vs. atomic O frequencies calculated from DFT. Modes shown include the *ν*_⊥_ stretch mode of O_2_ (blue circles), *δ*_⊥_ rotation of O_2_ (green triangles), and the O–O stretch (red squares). Frequencies of atomic oxygen are for the *ν*_⊥_ mode. DFT calculations are for adsorption on (100) and (111) fcc transition metal surfaces. Atomic O is at the most stable site in each case and O_2_ is at the bridge site parallel to the surface. Only the points corresponding to the *ν*_⊥_(M–O_2_) modes are labeled, as all DFT frequencies are for these metals. The slopes of the regressed lines are −2.19, 0.87, and 0.63 for the red, green, and blue lines, respectively. The intercepts are all positive

To support the mode assignments based on scaling of literature experimental frequencies, we performed our own DFT studies for molecular oxygen and atomic oxygen on the (111) and (100) facets of transition metal surfaces at their most stable site (Fig. 7) As expected, the *δ*_⊥_(M–O_2_) mode (Fig. 7; green triangles) correlates positively with the *ν*_⊥_(M–O) frequency. While the slopes determined from experimental data in Supplementary Fig. 8 are slightly lower than from DFT, the differences are well within uncertainty, lack of precise knowledge regarding the experimental adsorption site, and the infinite metal mass approximation. Of significance is that the experimental and computational 500–800 cm^−1^ frequencies scale positively with the *ν*_⊥_(M–O) frequency.

Our DFT-calculated ν(O–O) stretch (Fig. 7; red squares) shows a negative slope when scaled with the *ν*_⊥_(M–O) stretch, in direct contrast to the experimentally observed modes shown in Supplementary Fig. 8. As the DFT-calculated ν(O–O) frequency matches experiment for O_2_ on some metals, such as Pt(111), the deviations are most likely due to the presence of oxides in experiment on stronger binding metals^52^. Bond-order conservation^53^ rules and back donation of metal’s *d*-electrons to antibonding O_2_ orbitals^54^ both suggest that the ν(O–O) stretch should decrease with increasing *ν*_⊥_(M–O_2_) stretch^8, 27^. Thus, our DFT calculations and scaling experimental data support that the 500–800 cm^−1^ frequencies belong to frustrated rotations and that 600–1000 cm^−1^ frequencies observed experimentally are not purely due to an O–O stretching mode.

Vibrational scaling relations can also provide quantitative insight from complex surface spectra where the exact surface structure is unknown, making it difficult or impossible to replicate the spectra from DFT alone. Already, machine learning has turned FTIR and Raman spectroscopy into quantitative tools for detecting bulk concentrations and distinguishing between different strains of bacteria^55^. As an example from heterogeneous catalysis, previous DFT and experimental studies have shown that Ni-Pt-Pt (Ni on top of subsurface Pt, followed by core Pt) is very active for ammonia decomposition^3^. Experimental HREELS studies assigned the 494 cm^−1^ peak to both surface N and NH_2_ by comparing to known peaks on single crystal monometallics. Our scaling relations in Fig. 1b reveals that if the 494 cm^−1^ peak is assigned to chemisorbed atomic N, the frequency of NH_2_ should be 424 cm^−1^ and that of NH should be 469 cm^−1^. These NH_*X*_ scaling relations suggest that the 494 cm^−1^ peak should be attributed to a combination of N and NH whereas the 440 cm^−1^ peak cited in the paper should include contributions from NH_2_, in addition to the background CO. Given the vibrational peak of atomic N, scaling relations developed in this work can be used to determine the concentration of each species on the surface through spectral deconvolution^56^.

Finally, in combination with machine learning, the scalings developed herein may be valuable to characterize the surfaces of complex materials, like those of bimetallics. Such surfaces are non-ideal. Rather, they contain different terminations, e.g., patches of Ni on Pt adjacent to Pt patches of varying size, steps, and single point defects. We envision that the proposed scaling relations could, in conjunction with machine learning, be used to unravel atomistic structural detail from the spectra to complement imaging techniques whose operando use is still under development. Future work will be needed to fully explore this opportunity.

### Effect of VSRs on predicted thermochemistry

VSRs can be used to discern whether scaling vibrational contributions to Gibbs free energy (G_vib_) is important. In Supplementary Note 6 and Supplementary Figs. 9 and 10 we show that, for the range of normal modes found for chemisorbed CO, G_vib_ can be approximated with a first order Taylor expansion of the nonlinear terms resulting in a linear contribution to G_vib_ from individual vibrational modes. Because different normal modes within the same molecule scale both with each other and with adsorption energy, G_vib_ often correlates linearly with adsorption energy. For certain molecules, such as for CO, scaling of G_vib_ is significant. For example, at 400 K, G_vib_ for CO on Pd is over 0.20 eV higher than on Ag, impacting microkinetic model predictions by over three orders of magnitude.

**Fig. 8:**
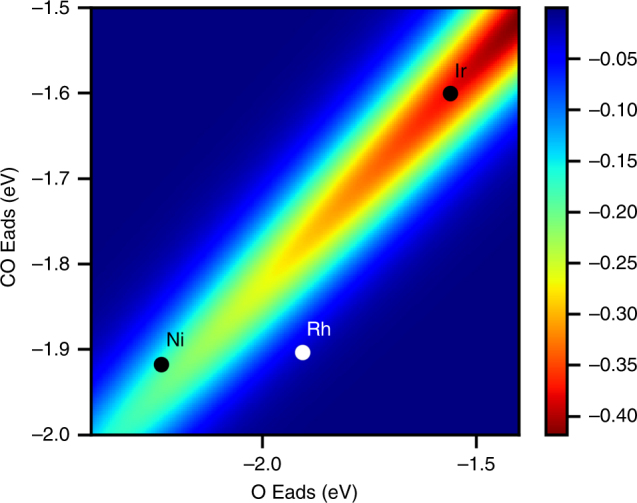
Error in predicted CO coverage. Error is without scaling of G_vib_ at 400 K, 1 bar and 100 ppm CO in O_2_ based on vibrational contributions for CO and O on Pd(111) at the fcc hollow site. *x* and *y* axes correspond to electronic adsorption energy calculated using the RPBE functional with D3 dispersion corrections. The color indicates the error in coverage, with the highest possible coverage of 1. Lack of vibrational scaling lowers the CO coverage

In Fig. 8 we report errors in carbon monoxide surface coverage for competitive adsorption of CO and O_2_, caused by neglect of G_vib_ scaling. The error in coverage is plotted for different binding energies of CO and O, as well as the positions with respect to the close-packed facet for Ir, Ni, and Rh on the binding energy map. The region where both CO and O_2_ are co-adsorbed in appreciable amounts is also the region where neglecting vibrational scaling effects produces the greatest error. This region is important because it is the catalytically active region for forming CO_2_ from CO and O_2_^57^. While coverage effects have not been included, the effects of scaling are expected to hold in the presence of lateral effects at higher coverages^58^. Clearly, accounting for G_vib_ scaling with frequencies is necessary to predict accurate coverages.

## Discussion

Surfaces contain an ensemble of chemical conformations, adsorption sites, and reaction pathways. Fortunately, vibrational spectra are mode specific and can achieve precision to within 1 cm^−1^. This precision often corresponds to less than 0.1 and 1% uncertainties for the gas and surface species, respectively^54^. Experimentally, frequencies can shift by a few percent over large temperature changes^59^. These shifts, however, result from changes in surface structure^60^ or co-adoption of other species. Parameter fitting to experimental gas-phase vibrational frequencies, rather than strictly using energies, has already improved DFT functional performance for predicting geometries beyond those achieved by CCSD(T) calculations^61^. While our calculated adsorbate frequencies were usually accurate to within 5%, the *ν*_⊥_ frequencies of adsorbed atomic nitrogen on Ni(111)^42^ and Cu(111)^38^ were higher than experiment by 15%. Spectroscopic studies on these surfaces therefore warrant more attention. Still, uncertainties in binding energies are often much larger than this^62^.

**Fig. 9:**
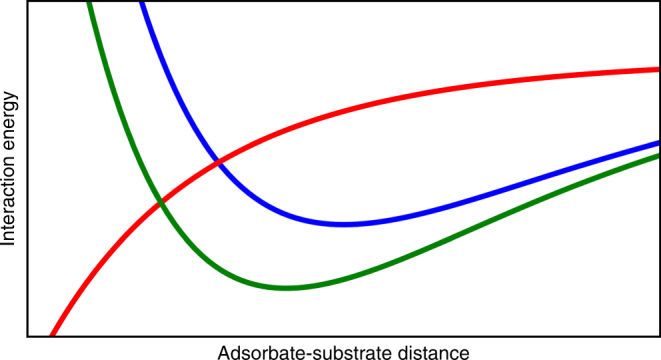
Interaction energy of metal orbitals with an adsorbate. The contributions from the *d*-band (Δ*E*_*d*_; red) and *sp*-band (Δ*E*_*sp*_; blue) combine to make the total adsorption energy (Δ*E*_ads_; green). The illustration is representative of interaction energies for atomic oxygen with the Pd(111) surface

Equation (8) implies that the relative error in *ν* is proportional to the square root of the sum of errors for *η*Δ*E*_*sp*_ and *R*_*n*_Δ*E*_*d*_. From standard error propagation, this is equivalent to half the relative error. Furthermore, if errors in Δ*E*_*sp*_ and Δ*E*_*d*_ are in the same direction (e.g., due to DFT-inherent overbinding)^63^, their contribution to the error in frequency partially cancel, since *η* is negative and *R*_*n*_ is positive, where *R*_*n*_ is given in Eq. (9). As a result, the error in frequency can be small, even when the error in adsorption energy is large. This cancellation of errors explains why DFT is usually able to predict vibrational frequencies accurately, despite errors in binding energies. A schematic depicting the competing nature of the *sp*-band and *d*-band contributions to binding energy, based on atomic oxygen on Pd(111)^64^, is shown in Fig. 9. The contribution from the *d*-band (Δ*E*_*d*_) and *sp*-band (Δ*E*_*sp*_) to adsorption energy (Δ*E*_ads_) is given in Eq. (2). The dependence of Δ*E*_*d*_ and Δ*E*_*sp*_ on distance were determined from LMTO and EMT, respectively.

Insights into the errors for vibrations driven by the adsorbate-metal interaction are important for several reasons. First, DFT frequencies can reliably be compared to experiment in order to determine binding sites. Not only are frequencies more accurate than adsorption energies, but they are sensitive to binding site as well. Second, the contribution to Gibbs energy from frequencies that scale across transition metals, as shown in the previous section, can confidently be included in microkinetic models. Accurate free energy scaling and microkinetic modeling calculations are essential in rapid screening of novel materials for catalyst discovery.

Both experimental data from literature and new DFT calculations reveal that vibrational frequencies of adsorbates scale both with each other and with adsorption energy on transition metal surfaces. Using effective-medium theory and linear muffin-tin orbital theory for adsorbate coupling to the transition metal *sp*-band and *d*-band, respectively, we derived vibrational scaling relations for modes driven by interaction between the adsorbate and metal surface. These VSRs result in correlations between the vibrational Gibbs energy of a species with the electronic adsorption energy, making them straightforward to include in kinetic and thermodynamic models and necessary for accurate thermochemistry predictions. We also showed that the competing nature of the *sp*-band and *d*-band can result in cancellation of errors, allowing accurate frequencies irrespective of the errors in adsorption energies. VSR-based contributions to thermochemistry should prove especially important for large molecules that contain a greater number of vibrational modes driven by interaction between the adsorbate and metal surface.

## Methods

### DFT calculations

We calculated binding energies and vibrational frequencies using the Vienna ab initio Simulation Package (VASP) version 5.4 with the projector augmented wave method (PAWs)^65^. We employ the RPBE density functional^66^ with D3 dispersion corrections^67^. Simulation methods were similar to those of our previous study^68^, including use of spin-polarized calculations for gas-phase species and ferromagnetic metals, a 3 × 3 × 1 Monkhorst-Pack k-point sampling grid for all slab calculations^69^, and a 400 eV plane-wave cutoff. The initial magnetic moments for each atom in spin-polarized calculations were set to VASP default values.

### Unit cell setup

For gas calculations, the supercell size was 10 × 10 × 10 Å. A Brillouin zone was sampled at the gamma point; a 0.005 eV/Å force cut-off was used in geometry optimizations. For slab calculations, the force cut-off was set to 0.02 eV/Å with 20 Å of vacuum space. The periodic cell consisted of four layers with 16 metal atoms in each layer; the bottom two layers were fixed at their bulk values, determined using a 15 × 15 × 15 k-point grid using the tetrahedron method with Blöchl corrections. Bulk metal lattice constants were pre-optimized with DFT using the Birch–Murnaghan equation of state^70^. All input files were created using the Atomistic Simulation Environment (ASE).

### Adsorption sites and reference

We study adsorption at the fcc hollow, bridge, and atop sites of (100), (110), and (111) transition metal facets. The adsorption energy is calculated according to

18 $${\mathrm{\Delta }}E_{{\mathrm{ads}}} = E_{\left( {{\mathrm{slab/adsorbate}}} \right)} - \left( {E_{{\mathrm{slab}}} + E_{{\mathrm{gas}}}} \right)$$

where the adsorption energy (Δ*E*_ads_) is determined from the energies of the combined adsorbate-slab system (Energy_(slab/adsorbate)_), the slab (*E*_slab_), and the gaseous adsorbate (*E*_gas_).

Perpendicular (*ν*_⊥_) and parallel (*ν*_||_) vibrations (see Supplementary Fig. 12) are identified based on the criteria developed in the Supplementary Methods.

### Data availability

The authors declare that all data supporting the findings of this study are available within the Article and its Supplementary Information files. An excel file is also provided that contains frequencies, energies, and other values calculated from DFT used in generating the figures contained in this work. Any other data will be provided by the corresponding author upon request.

## Electronic supplementary material

Supplementary Information

Description of Additional Supplementary Files

Supplementary Data 1
