# Supplementary material for: Scaling relationships and theory for vibrational frequencies of adsorbates on transition metal surfaces
Source: Nat Commun. 2017 Nov 29;8:1842. doi: 10.1038/s41467-017-01983-6 (PMC5705602; doi:10.1038/s41467-017-01983-6)
Supplement: Supplementary file 1 — Supplementary Information [file 41467_2017_1983_MOESM1_ESM.pdf]

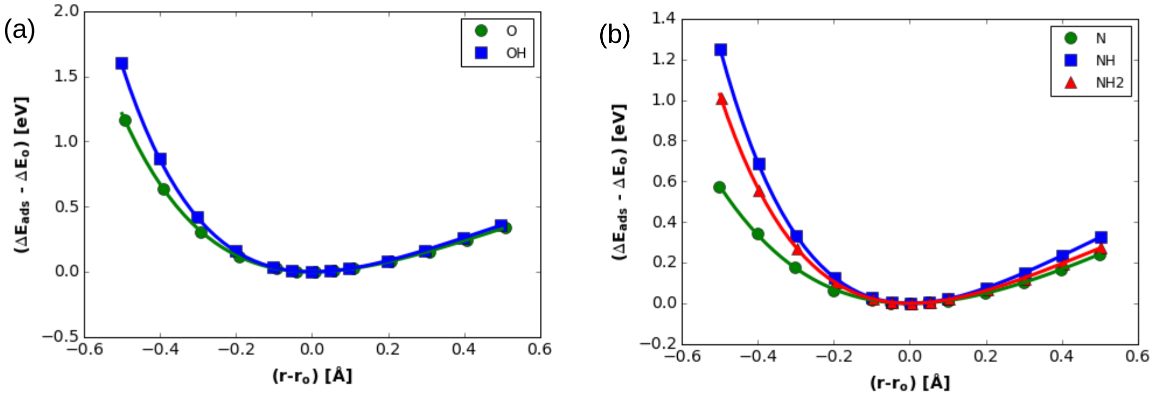

Supplementary Figure 1 | Adsorption energy fit to a Morse potential. (a) OH_X species on K(110) at the atop site. (b) NH_X species on K(110) at the atop site.

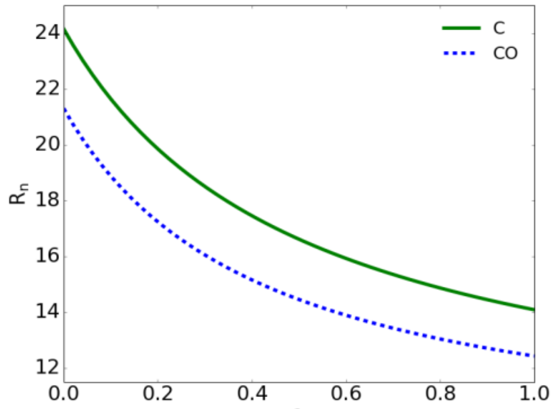

Supplementary Figure 2 | R_n vs. f_s. C (green, solid) and CO (blue, dashed). For both species, the distance is taken from the respective carbon atom and the nearest neighbor metal at the atop site of Pt(111).

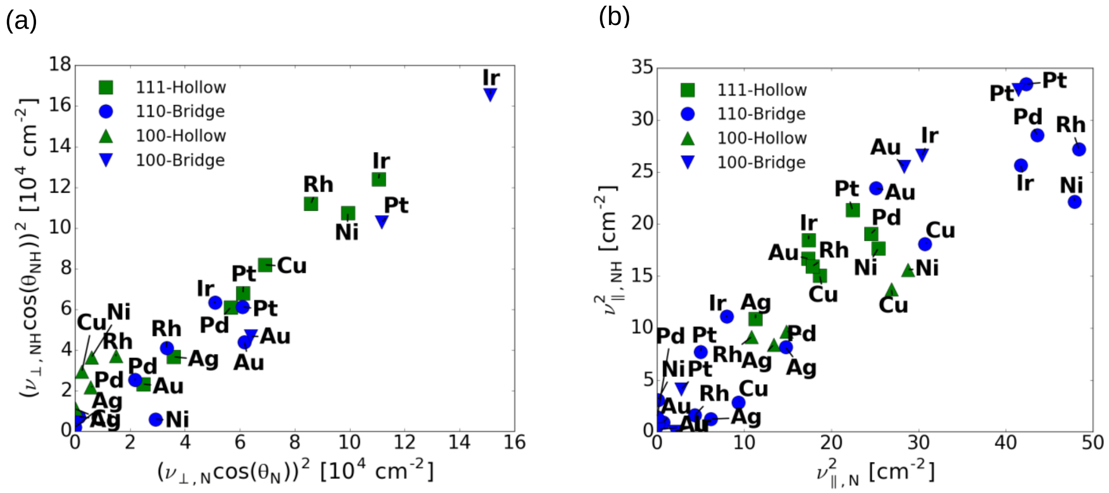

Supplementary Figure 3 | Frequency scaling for N vs. NH. Fits for (a) and (b) have slopes of 0.96 and 0.61.

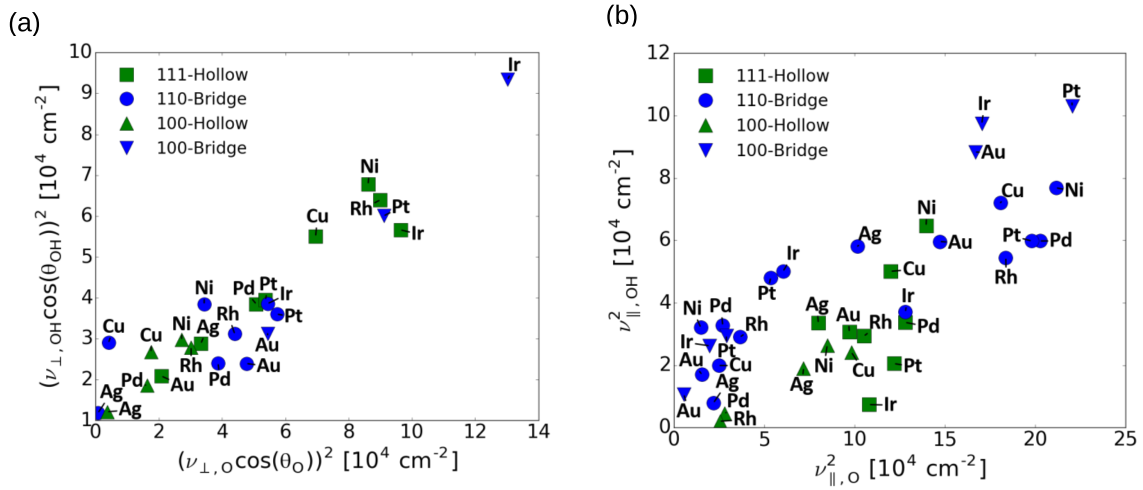

Supplementary Figure 4 | Frequency scaling for O vs. OH. Fits for (a) and (b) have slopes of 0.55 and 0.31.

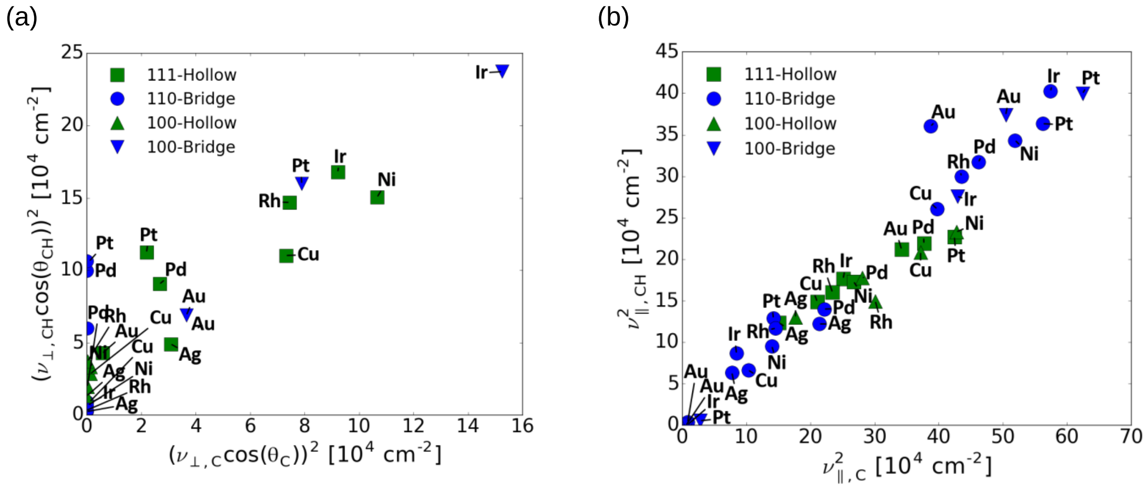

Supplementary Figure 5 | Frequency scaling for C vs. CH. Fits for (a) and (b) have slopes of 1.35 and 0.65.

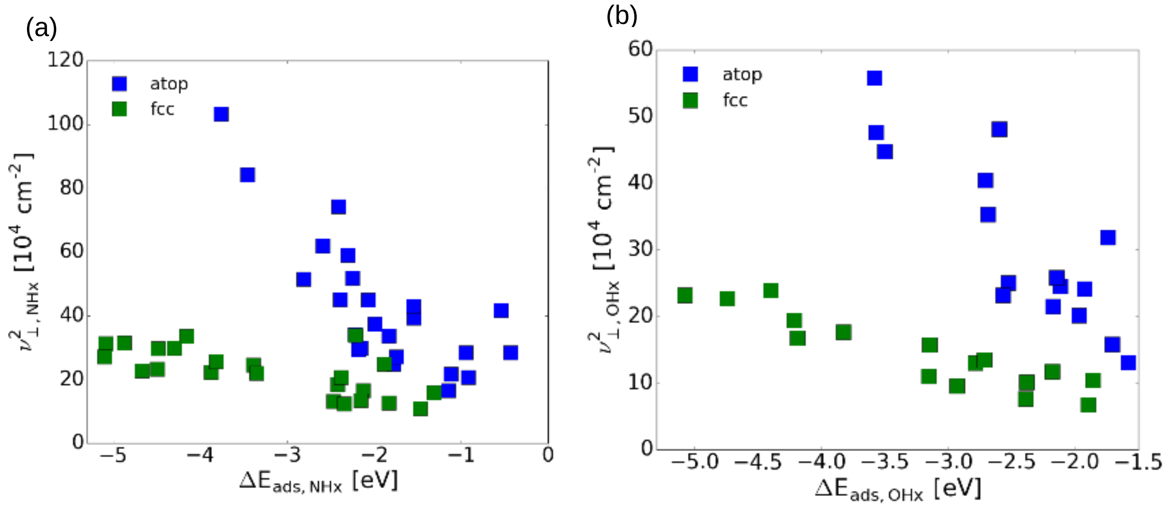

Supplementary Figure 6 | Scaling of $v_\perp$ frequencies with electronic adsorption energy. Values are from DFT for NHx and OHx species, x ≥ 0. Included are modes at atop sites (blue) and fcc sites (green). Frequencies shown: (a) NH_2, NH, and N, and (b) OH and O.

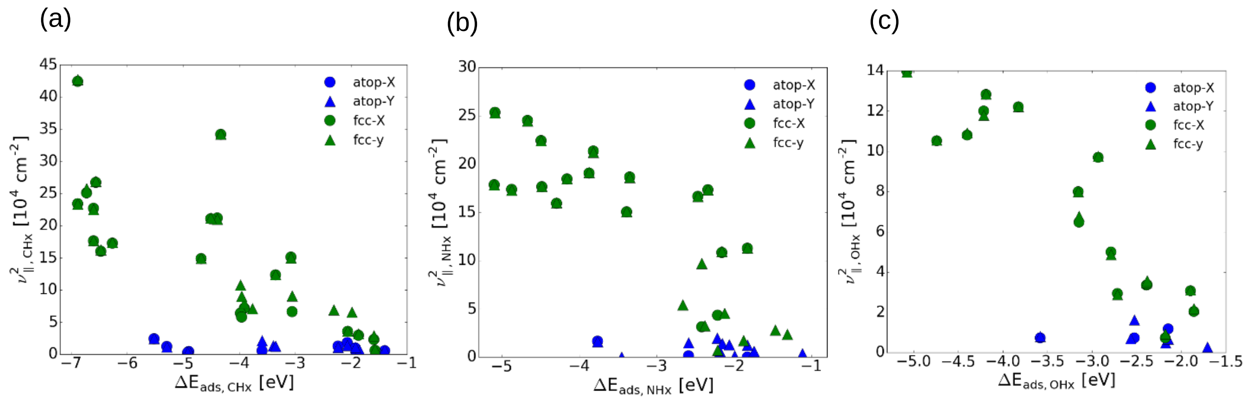

Supplementary Figure 7 | Scaling of $v_{||}$ frequencies with electronic adsorption energy. Values are from DFT for CHx, NHx and OHx species where x is ≥ **0**. Included are modes at atop sites (blue) and fcc sites (green). Frequencies shown: (a) CH_3, CH_2, CH, and C, (b) NH_2, NH, and N, and (c) OH and O.

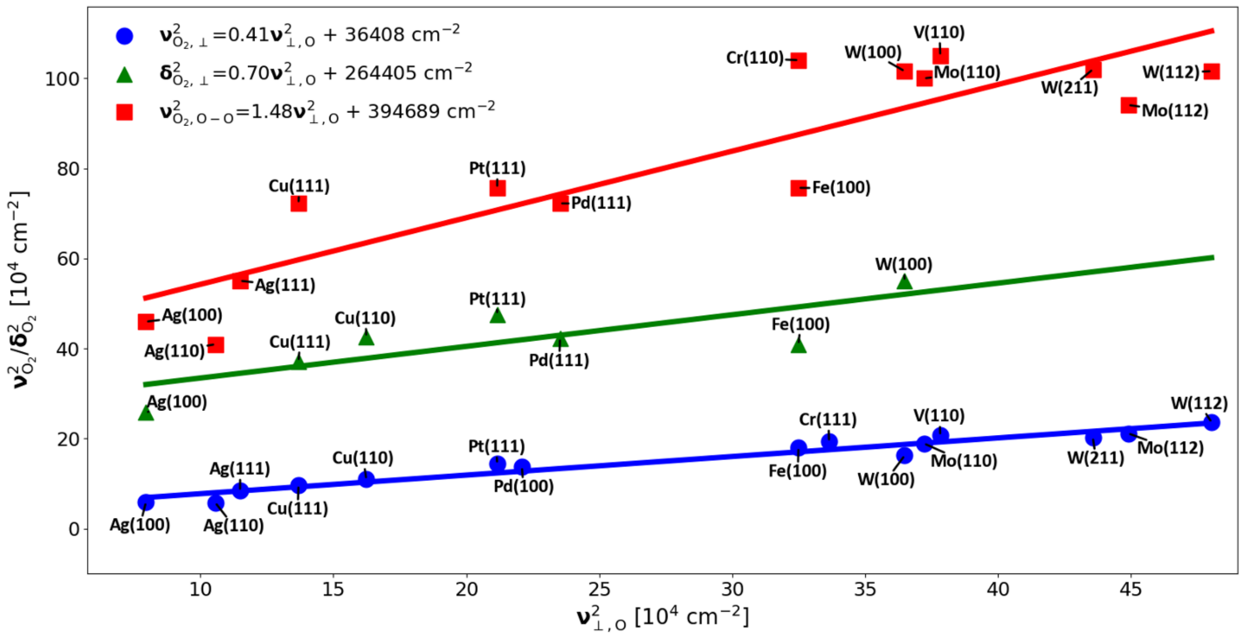

Supplementary Figure 8 | Scaling of O_2 frequencies with the $\nu_\perp$ atomic O frequencies. All frequencies are from experiment. Modes shown include the $\nu_\perp$ stretch mode of O_2 (blue circles), $\delta_\perp$ rotation of O_2 (green triangles), and the O-O stretch (red squares). Labels indicate the metal surface.

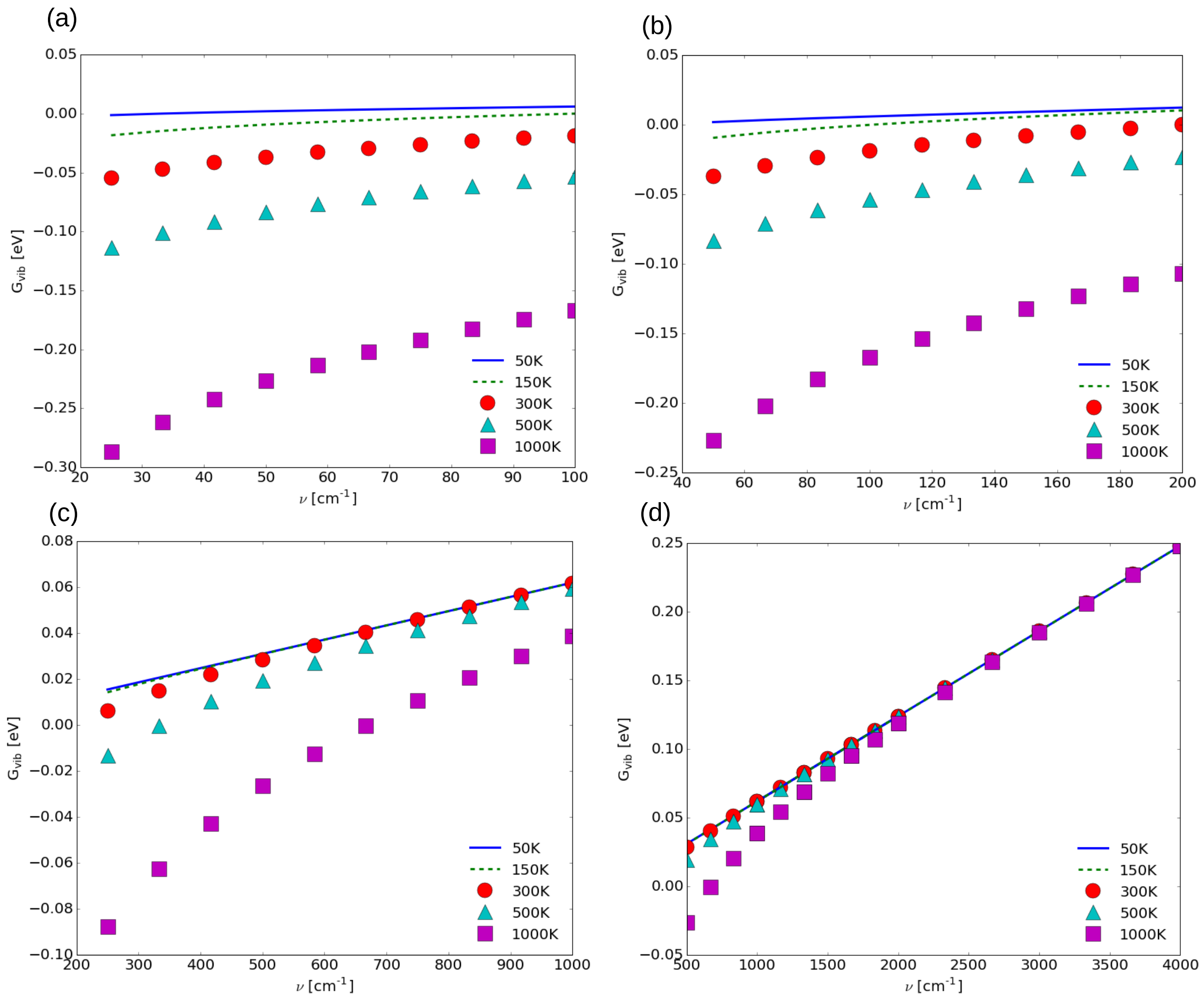

Supplementary Figure 9 | G_{vib} calculated for various frequencies. Temperatures are at 50 (blue line), 150 (green dash), 300 (red circles), 500 (cyan triangles), and 1000 (magenta squares) Kelvin. All frequencies are calculated according to 41.

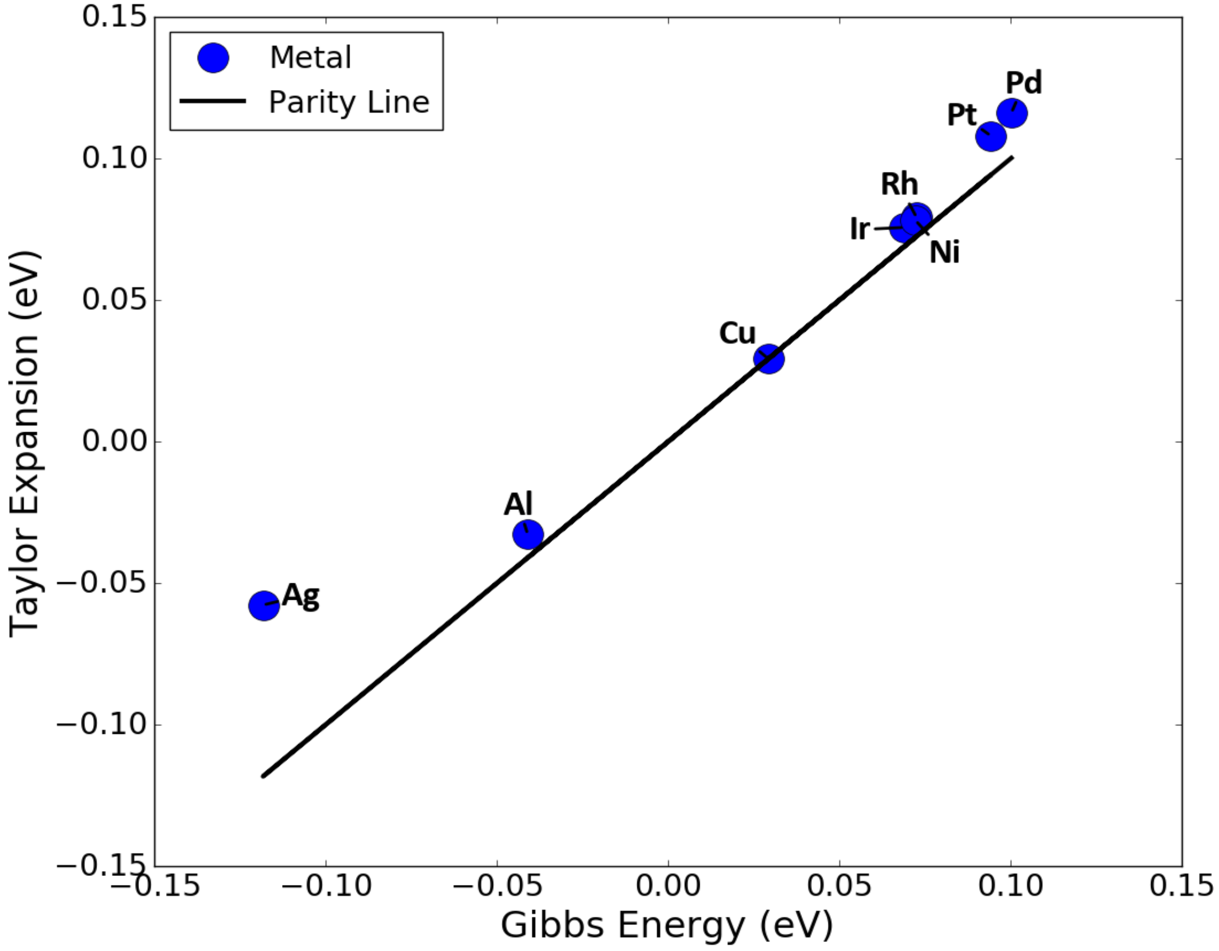

Supplementary Figure 10 | Vibrational Gibbs energy of CO on several 111 metals. Adsorption is at the fcc hollow site of the close packed surface. The x-axis is the Gibbs energy at 400 K under the harmonic approximation and the y-axis is the first order Taylor series approximation of this Gibbs Energy. Frequencies were calculated from normal mode analysis of the potential energy surface defined using the RPBE functional with D-3 dispersion corrections in VASP.

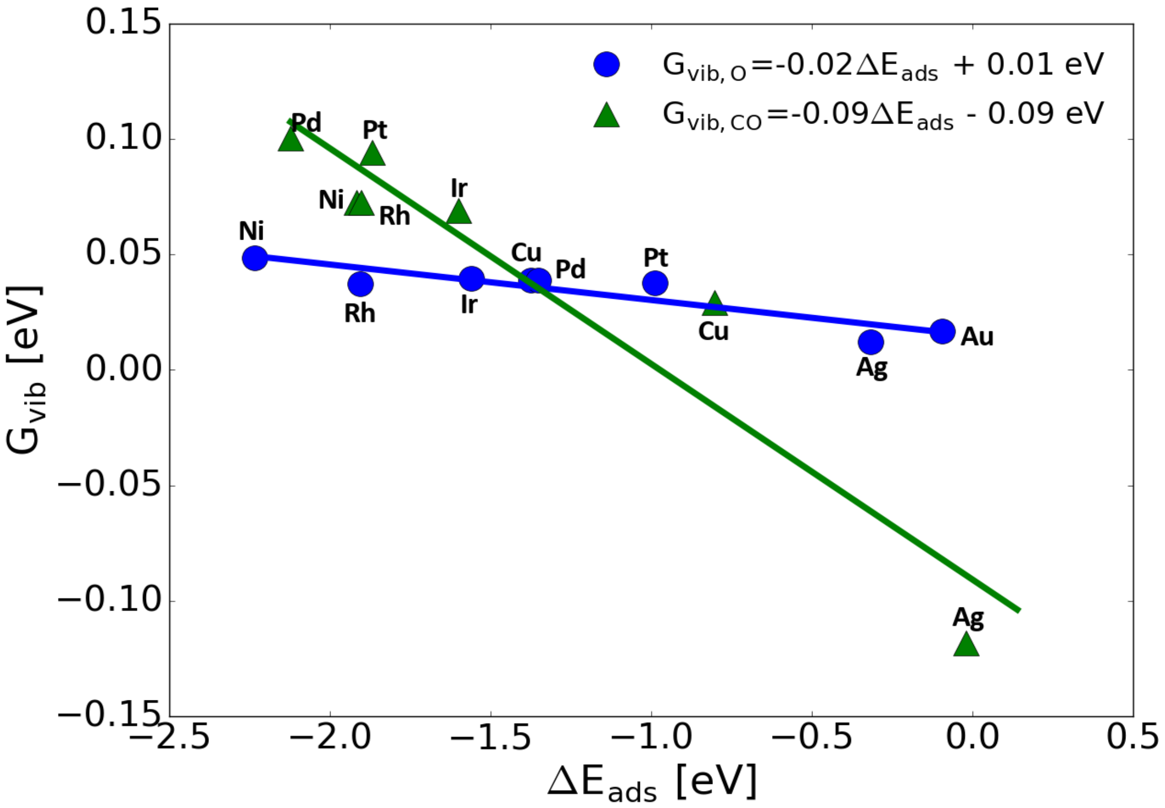

Supplementary Figure 11 | Scaling of vibrational Gibbs energy with the adsorption energy. Only the electronic portion of adsorption energy is shown and vibrational Gibbs energy is at 400 K based on the harmonic approximation. The y-axis is the vibrational contribution to Gibbs energy and the x-axis is the electronic portion of the adsorption energy as determined from DFT.

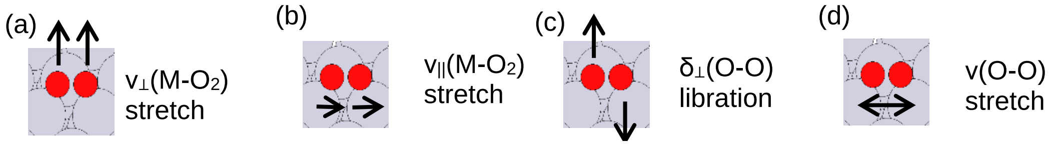

Supplementary Figure 12 | Normal Modes of O2 (red) adsorbed on a metal surface (gray). Modes shown: (a) Metal-oxygen stretch mode perpendicular to the surface; (b) Metal-oxygen stretch mode parallel to the surface; (c) Frustrated rotation; (d) Internal oxygen-oxygen stretch mode parallel to the surface.

Supplementary Table 1 Equilibrium bond lengths. Values are for AHx adsorbates at the atop site of (111) surfaces according to RPBE with D3 dispersion corrections (Angstroms).

Metal	O	OH	N	NH	NH_2	C	CH	CH_2	CH_3
Ag	1.97	2.11	1.94	2.03	2.10	1.95	1.96	2.04	2.20
Au	1.93	2.1	1.87	1.98	2.05	1.87	1.94	1.98	2.13
Cu	1.75	1.86	1.73	1.75	1.87	1.77	1.74	1.84	2.01
Ir	1.79	2.00	1.68	1.83	1.95	1.72	1.73	1.88	2.13
Ni	1.67	1.8	1.64	1.68	1.81	1.65	1.65	1.77	1.95
Pd	1.81	1.98	1.74	1.86	1.94	1.87	1.88	1.87	2.05
Pt	1.82	1.99	1.73	1.86	1.94	1.73	1.75	1.87	2.07
Rh	1.77	1.98	1.66	1.77	1.93	1.68	1.71	1.85	2.09

Supplementary Table 2 Projected density of states. Orbital occupancies are for the bonding atom of the adsorbate at the fcc-hollow site of Pt(111) using VASP LORBIT=11.

Adsorbate	Total Charge	s	p	f_s	f_p
C	2.343	0.935	1.408	0.399	0.601
CH	2.39	0.875	1.515	0.366	0.634
CH2	2.428	0.844	1.584	0.348	0.652
CH3	2.494	0.827	1.667	0.332	0.668
N	3.533	1.325	2.208	0.375	0.625
NH	3.688	1.243	2.445	0.337	0.663
NH2	3.847	1.237	2.61	0.322	0.678
O	4.9	1.62	3.28	0.331	0.669
OH	5.084	1.579	3.505	0.311	0.689

Supplementary Note 1 - Derivation of equation 11 in the main text

The main text discusses evidence and theory for the scaling of frequencies of AHx species with the frequencies of their atomic adsorbates, A, across transition metal surfaces. The following derivation is based on the sp and d-band contributions to adsorption energy scaling relations (LSRs) on transition metal surfaces.[1, 2] Frequency scaling predictions are shown in Figure 3 of the main text. The harmonic approximation gives

$$v = \frac{1}{2\pi}\sqrt{\frac{k}{\mu}} \qquad 1$$

where the frequency, v, is a function of a force constant (k) and the reduced mass of a vibrating system (μ). The force constant is the second derivative of energy $V(r)$, or adsorption energy $\Delta E(r)$, with respect to the normal mode displacement r_{norm}

$$k = \frac{\partial^2\big(\Delta E(r_{norm})\big)}{\partial r_{norm}^2}. \qquad (2)$$

For an adsorbate on transition metal surfaces, we can separate adsorption energy into contributions from hybridization of the adsorbate orbitals with the metal sp and d-bands according to 3,

$$\Delta E = \Delta E_{sp} + \Delta E_d \qquad (3)$$

where ΔE_{sp} and ΔE_d represent contributions to the binding energy from the adsorbate interacting with the metal sp and d-band, respectively.[1]

Because the adsorption energy is additive, we can redefine the force constant as the sum of sp and d coupling contributions:

$$k = k_{sp} + k_d \qquad (4)$$

$$k_{sp} = \frac{\partial^2\big(\Delta E_{sp}\big)}{\partial r_{norm}^2} \qquad (5)$$

$$k_d = \frac{\partial^2(\Delta E_d)}{\partial r_{norm}^2} \qquad (6)$$

where k_{sp} and k_d are contributions to the force constant from metals sp and d-band, respectively.

Because the metal sp electrons are delocalized, they can be treated as a homogenous electron gas, making Effective Medium Theory (EMT) ideal for modeling their interaction with the adsorbate.[3, 4] It is well known that electron density around an adsorbate varies exponentially with distance for an atom embedded in a homogenous electron gas surface.[5, 6] It has also been shown computationally, and supported experimentally on Al, that the energy of He varies linearly with electron density.[7] A sum of exponential functions has been applied successfully in neural networks for fitting potential energy surfaces to atomic distances.[8, 9] Therefore ΔE_{sp} can then be taken as an exponential function of distance between the adsorbate and transition metal atoms.

As many exponential functions as needed may be summed to approximate ΔE_{sp}. A modified sum of exponentials is expressed in Supplementary Equation 7

$$\Delta E_{sp} = \Delta E_{sp,o}\left(a - \sum_{i=1}^{n} b_i \exp^{c_i(r-r_o)}\right) \qquad (7)$$

where a, b_i, c_i, and n are adsorbate dependent constants, $(r - r_o)$ is the separation distance from equilibrium and $\Delta E_{sp,o}$ is the binding energy at equilibrium. The distance, r, is between the adsorbate atom closest to the surface and the nearest neighbor metal atom. The distance, r_o, is the distance between the adsorbate and metal atom at equilibrium. For perpendicular frequencies at the atop site, $r = r_{norm}$. For other modes and adsorption sites, the developed theory can by extended such that $r = g(\theta)r_{norm}$ where θ is often the angle between r_{norm} and r.

It is then straightforward to express k_{sp} as a function of ΔE_{sp}:

$$k_{sp} = -\Delta E_{sp,o}\sum_{i=1}^{n} b_i c_i^2 \exp^{c_i(r-r_o)} \qquad (8)$$

and when $r = r_o$,

$$k_{sp} = \eta \Delta E_{sp} \quad 9$$

where,

$$\eta = -\sum_{i=1}^{n} b_i c_i^2 \quad 10$$

Alkali, alkaline earth, and aluminum closely resemble a homogeneous electron gas, or jellium.[10, 11] Like transition metals in their bulk state, potassium also has a half-filled outer s-orbital.[12] For this reason, the adsorption energies for OHx and NHx adsorbates on a K(110) surface were fit to a Morse potential ($n = 2$ and $a = 0$) to demonstrate the exponential dependency of ΔE_{sp} on distance. The results for the fits are shown in Supplementary Figure 1, supporting the functional form of ΔE_{sp} and thus Supplementary Equation 9.

Under the Linear Muffin Tin Orbital (LMTO) theory of Anderson, ΔE_d for an adsorbate described by a single azimuthal quantum number is given by Supplementary Equation 11,

$$\Delta E_d = \alpha \left(\frac{M_A M_M}{r^m} \right)^2 \quad 11$$

where α is a proportionality constant, M_A and M_M are constants given by the adsorbate and metal separately, r is the equilibrium distance between the adsorbate and nearest neighbor metal atom, and m is determined by the azimuthal quantum numbers of the interacting adsorbate bonding orbital and metal d states, l_a and l_d, respectively as shown in Supplementary Equation 12.[4, 13, 14]

$$m = (l_a + l_d + 1) \quad 12$$

When the adsorbate electrons interacting with the metal electrons are in the s state, $l_a = 0$ and $m = 3$. When these interacting adsorbate electrons are in the p-state, $l_a = 1$ and $m = 4$. We simplify Supplementary Equation 11 in Supplementary Equation 13,

$$\Delta E_d = \frac{\beta}{r^n} \quad 13$$

where $\beta = \alpha (M_A M_M)^2$ and $n = 2m$.

The contribution to the force constant from the d-band coupling is found by differentiation:

$$\frac{\partial(\Delta E_d)}{\partial \mathrm{r}} = -n \frac{\beta}{r^{n+1}} \quad 14$$

$$\frac{\partial^2(\Delta E_d)}{\partial \mathrm{r}^2} = n(n+1) \frac{\beta}{r^{n+2}} \quad 15$$

$$k_d = \frac{n(n+1)}{r^2} \Delta E_d \quad 16$$

As most adsorbates of interest have hybridized s and p states, their total contribution to ΔE_d will be additive according to Supplementary Equation 17,

$$\Delta E_d = \beta \left(\frac{f_s}{r^{n_s}} + \frac{f_p}{r^{n_p}} \right) \quad 17$$

where f_s and f_p are related to the s and p character of adsorbate orbital that bonds to the metal atom with $n_s = 6$ and $n_p = 8$, respectively. In this work, f_s and f_p are estimated from the total number of s and p electrons in the adsorbate atom, A, that bonds with the metal surface. Supplementary Table 2 contains the density of states projected onto the bonding adsorbate atom and shows that the p character of the bonding atom increases only slightly with hydrogenation. The force constant is then found accordingly:

$$\frac{\partial(\Delta E_d)}{\partial \mathrm{r}} = -\beta\left(\frac{n_s f_s}{r^{n_s+1}} + \frac{n_p f_p}{r^{n_p+1}}\right) \tag{18}$$

$$\frac{\partial^2(\Delta E_d)}{\partial \mathrm{r}^2} = \beta\left(\frac{n_s(n_s+1)f_s}{r^{n_s+2}} + \frac{n_p(n_p+1)f_p}{r^{n_p+2}}\right) \tag{19}$$

$$k_d = \Delta E_d\left(\frac{f_s}{r^{n_s}} + \frac{f_p}{r^{n_p}}\right)^{-1}\left(\frac{n_s(n_s+1)f_s}{r^{n_s+2}} + \frac{n_p(n_p+1)f_p}{r^{n_p+2}}\right) \tag{20}$$

Upon simplifying Supplementary Equation 20,

$$k_d = \Delta E_d\left(\frac{r^{n_s}r^{n_p}}{f_s r^{n_p} + f_p r^{n_s}}\right)\left(\frac{n_s(n_s+1)f_s}{r^{n_s+2}} + \frac{n_p(n_p+1)f_p}{r^{n_p+2}}\right) \tag{21}$$

resulting in Supplementary Equation 22,

$$k_d = \Delta E_d \frac{f_s n_s(n_s+1)r^{n_p} + f_p n_p(n_p+1)r^{n_s}}{r^2(f_s r^{n_p} + f_p r^{n_s})} \tag{22}$$

Terms in Supplementary Equation 22 will be grouped together according to Supplementary Equation 23 as a single distance-dependent term, R_n.

$$R_n = \frac{f_s n_s(n_s+1)r^{n_p} + f_p n_p(n_p+1)r^{n_s}}{r^2(f_s r^{n_p} + f_p r^{n_s})} \tag{23}$$

The frequency of adsorbate A is given by making appropriate substitutions.

$$\nu^A = \frac{1}{2\pi}\sqrt{\frac{k_{sp}^A + k_d^A}{\mu^A}} \tag{24}$$

$$\nu^A = \frac{1}{2\pi\sqrt{\mu^A}}\sqrt{\eta^A \Delta E_{sp}^A + \Delta E_d^A R_n^A} \tag{25}$$

where the superscript A refers to a property pertaining to adsorbate A.

The scaling of energy between two adsorbates (LSR) is determined by coupling between adsorbate orbitals and the metal d-band, resulting in a linear relationship between the d-couplings of two adsorbates on transition metal surfaces.[1] The slope of the energy LSR is shown in 26,

$$\Delta E_d^{AHx} = m_E \Delta E_d^A \tag{26}$$

where the subscript AHx indicates a property relating to the hydrogenated form of adsorbate A, and m_E is the slope of the LSR. Deviations in the LSR slope due to dispersion corrections are negligible for the adsorbates considered

here. The slope of the LSR is independent of ΔE_{sp} because the coupling of the adsorbate to the metal sp-band is constant across all transition metals.[1, 12]

The frequency for adsorbate AHx is then given in Supplementary Equation 27

$$\nu^{AHx} = \frac{1}{2\pi\sqrt{\mu^{AHx}}}\sqrt{\eta^{AHx}\Delta E_{sp}^{AHx} + m_E \Delta E_d^A R_n^{AHx}}. \tag{27}$$

The slope of the frequency scaling relation (VSR) is defined as the first derivative of ν^{AHx} with respect to ν^A and derived in the following steps.

First, ΔE_d^A can be solved as a function of ν^A in 25 according to Supplementary Equation 28

$$\Delta E_d^A = \left(4\pi^2\mu^A(\nu^A)^2 - \eta^A \Delta E_{sp}^A\right)(R_n^A)^{-1} \tag{28}$$

The result of Supplementary Equation 28 is substituted into Supplementary Equation 27 and the partial derivative is found:

$$\nu^{AHx} = \frac{1}{2\pi\sqrt{\mu^{AHx}}}\sqrt{\eta^{AHx}\Delta E_{sp}^{AHx} + m_E\left(4\pi^2\mu^A(\nu^A)^2 - \eta^A\Delta E_{sp}^A\right)(R_n^A)^{-1}R_n^{AHx}} \tag{29}$$

$$\frac{\partial \nu^{AHx}}{\partial \nu^A} = \frac{\frac{1}{2} * 2m_E(4\pi^2\mu^A\nu^A)\ (R_n^A)^{-1}R_n^{AHx}}{2\pi\sqrt{\mu^{AHx}}\sqrt{\eta^{AHx}\Delta E_{sp}^{AHx} + m_E\left(4\pi^2\mu^A(\nu^A)^2 - \eta^A\Delta E_{sp}^A\right)(R_n^A)^{-1}R_n^{AHx}}}. \tag{30}$$

Further simplification is made by substituting Supplementary Equation 25 for ν^A such that

$$\frac{\partial \nu^{AHx}}{\partial \nu^A} = m_E\sqrt{\frac{\mu^A}{\mu^{AHx}}}\left(\frac{R_n^{AHx}}{R_n^A}\right)\frac{\sqrt{\eta^A\Delta E_{sp}^A + \Delta E_d^A R_n^A}}{\sqrt{\eta^{AHx}\Delta E_{sp}^{AHx} + m_E\Delta E_d^A R_n^{AHx}}}. \tag{31}$$

The slope of the scaling relation is related to the total derivative and given by:

$$\frac{d\nu^{AHx}}{d\nu^A} = \frac{\partial\nu^{AHx}}{\partial\nu^A} + \frac{\partial\nu^{AHx}}{\partial \mathrm{R}_\mathrm{n}^{AHx}}\frac{d\mathrm{R}_\mathrm{n}^{AHx}}{d\nu^A} + \frac{\partial\nu^{AHx}}{\partial\mathrm{R}_\mathrm{n}^A}\frac{d\mathrm{R}_\mathrm{n}^A}{d\nu^A} + \frac{\partial\nu^{AHx}}{\partial\eta^{AHx}}\frac{d\eta^{AHx}}{d\nu^A} + \frac{\partial\nu^{AHx}}{\partial\eta^A}\frac{d\eta^A}{d\nu^A}. \tag{32}$$

We note that

$$\frac{\partial\nu^{AHx}}{\partial\eta^{AHx}} \cong -\frac{\partial\nu^{AHx}}{\partial\eta^A} \tag{33}$$

as

$$\Delta E_{sp}^{AHx} \cong m_E\Delta E_{sp}^A(R_n^A)^{-1}R_n^{AHx}. \tag{34}$$

As discussed extensively in literature and mentioned earlier, the equilibrium bond length is determined primarily by the coupling of the adsorbate to the metal sp-band.[1] For this reason, r varies much less than ν^A across transition

metals such that $\frac{d\mathrm{R}_n^A}{d\nu^A}$ is negligible, as shown in Supplementary Table 1, and the partial derivative of ν^{AHx} with respect to ν^A is a very good approximation for the total derivative.

$$\frac{d\nu^{AHx}}{d\nu^A} = m_E \frac{\mu^A}{\mu^{AHx}} \left(\frac{R_n^{AHx}}{R_n^A}\right) \frac{\nu^A}{\nu^{AHx}} \qquad 35$$

Finally, by integration we get the following relation:

$$\frac{d([\nu^{AHx}]^2)}{d([\nu^A]^2)} = m_E \frac{\mu^A}{\mu^{AHx}} \left(\frac{R_n^{AHx}}{R_n^A}\right). \qquad 36$$

For normal modes where $r \neq r_{norm}$, we have

$$\frac{d([g^{AHx}(\theta)\nu^{AHx}]^2)}{d([g^A(\theta)\nu^A]^2)} = m_E \frac{\mu^A}{\mu^{AHx}} \left(\frac{R_n^{AHx}}{R_n^A}\right). \qquad 37$$

For the $\nu_\perp$ at hollow and bridge sites, $g(\theta) = \cos(\theta)$, where θ is the angle between the perpendicular normal mode displacement of the adsorbate and the nearest neighbor metal atom, is a good approximation.

It is possible for f_s to vary drastically from one adsorbate to another when the bonding atom is the same as demonstrated for C vs. CO in Supplementary Figure 2. The nonbonding electrons localized to the carbon atom of CO have mostly p character while the sigma-bonding electron has mostly s character.[15] If the lone pair and pi-orbital on the carbon atom is primarily responsible for CO bonding with the metal surface, f_s for CO should be close to zero. Atomic carbon, on the other hand, should have significant s character in its interaction with the metal surface. The orbital fractions in Supplementary Table 2 were calculated from the charge contributed by the s and p orbitals according to VASP's projected density of states analysis.

Supplementary Note 2 - Derivation of equation 13 in the main text

The derivation of the VSR intercept, b_v, is relatively straightforward. The frequency of an adsorbate, A, is zero when the contributions from coupling to the sp-band and d-band cancel out such that

$$\Delta E_d^A R_n^A = -\eta^A \Delta E_{sp}^A \qquad 38$$

and

$$\Delta E_d^A = -\frac{\eta^A \Delta E_{sp}^A}{R_n^A}. \qquad 39$$

Inserting Supplementary Equation 38 into Supplementary Equation 27 then results in Supplementary Equation 40 (Equation 14 in the main text),

$$b_v = \frac{1}{2\pi\sqrt{\mu^{AHx}}} \sqrt{\eta^{AHx} \Delta E_{sp}^{AHx} - m_E \eta^A \Delta E_{sp}^A \frac{R_n^{AHx}}{R_n^A}} \qquad 40$$

The intercepts of the VSR, developed here, and the LSR, from literature, are similar in that they both depend on the sp-band contribution to the binding energy and the LSR slope. The VSR intercept also depends on the reduced mass of the system, the exponential constants from Supplementary Equation 10, and the radial dependence from Supplementary Equation 23.

Supplementary Note 3 - Frequency scaling, hollow sites and parallel frequencies

Perpendicular and parallel frequency scaling (VSRs) for A vs. AH species at sites not shown in the main text can be found in Supplementary Figure 3-5. All (111) and (100) surface data points are for adsorption at the site that best stabilizes atomic oxygen. On the (110) surface, all DFT calculations were for adsorption at the long bridge site as this is generally the most stable for atomic adsorbates. Panels (a) and (b) indicate perpendicular and parallel frequencies for adsorption, respectively, at the following sites: (111)-Hollow (green squares), (110)-Long Bridge (blue circles), (100)-Hollow (green triangles), and the (100)-Bridge (blue upside down triangles). While the VSRs presented here have more scatter due to greater surface geometry dependence, the slopes of the VSRs are similar to those found in the main text. For bridge sites, both non-degenerate parallel frequencies are included. The $\cos(\theta)$ term has been included in scaling of the $\nu_{\perp}$ frequencies but not the $\nu_{||}$ frequencies. An Excel file containing of all energy and frequencies used to generate these figures is supplied along with this supporting information.

There are several reasons for the larger variance in the VSR slopes at hollow and bridge sites then the atop sites as shown in the main paper: (1) $g(\theta)$ is not truly constant with respect to r_{norm} and ν^{A}, (2) R_n^{AHx}/R_n^{A} varies more across metals, and (3) there is greater variance in the LSR slope.

A model that accounts for more complete geometric information should be able to better capture $\nu_{\perp}$ modes and parallel frequencies at hollow and bridge sites. For example, on the (100) and (110) surfaces second nearest neighbor metal atoms, below the adsorbate, are often close enough to the adsorbate to have a significant effect on the $\nu_{\perp}$ frequency. For this same reason, the nearest neighbor metals atoms to carbon are actually different when CH, as opposed to atomic carbon, is adsorbed on either the Pt(111) and Pd(111) surface. Because the nearest neighbor metal atoms lie on almost the same xy-plane as the adsorbate atom at some bridge and hollow sites, the $\nu_{||}$ modes scale with less error than the $\nu_{\perp}$ modes at those sites.

Supplementary Note 4 - Scaling of frequencies with adsorption energy

The main text already covers scaling of $\nu_{\perp}$ frequencies with adsorption energies at the atop and fcc sites for CHx species. Supplementary Figure 6 shows $\nu_{\perp}$ scaling with energy for NHx and OHx species. Supplementary Figure 7 depicts scaling of $\nu_{||}$ DFT-calculated frequencies for CHx (a) NHx (b), and OHx (c), and C, N, and O, against electronic adsorption energies. Adsorption is at the fcc-hollow (green data points) and atop site (blue data points) of the close-packed surface of FCC metals. Only frequencies corresponding to a concave potential energy surface in the direction of the normal mode displacement (non-imaginary) have been included; thus relatively few $\nu_{||}$ frequencies at atop sites are shown.

Supplementary Note 5 - Scaling of experimental molecular and atomic oxygen frequencies

Scaling frequencies can aid in interpreting complex experimental spectra. We demonstrate this benefit by scaling experimental O_2 and O frequencies in Supplementary Figure 8[16-33], and comparing to DFT results. Experimental studies of molecular oxygen agree that frequencies between 200 and 400 cm^{-1} correspond to $\nu_{\perp}$(metal-O_2) stretches (Supplementary Figure 8; blue circles).[21, 24, 32] The modes between 500 and 800 cm^{-1} (Supplementary Figure 8; green triangles) are usually cast as internal, parallel to the surface O-O stretches of molecular species adsorbed at a bridge site.[21, 32, 34, 35] Frequencies between 600 and 1000 cm^{-1} (Supplementary Figure 8; red squares) are often attributed to O-O stretches of a molecular species at an atop site.

Supplementary Note 6 - Effect of vibrations on Gibbs Energy and coverage at equilibrium

Under the harmonic oscillator assumption, vibrational contributions to Gibbs Energy take the form 41,

$$G_{vib} = \frac{1}{2}h\nu + k_B T\, ln\left(1 - e^{\frac{-h\nu}{k_B T}}\right) \qquad 41$$

where ν is a fundamental mode, h is the Planck constant, k_B is the Boltzmann constant, and T is the temperature.[36] In total, the vibrational Gibbs energy (G_{vib}) includes the zero point energy, entropy, and all temperature dependent enthalpic contributions.

The nonlinear term in 41 can be approximated as linear under the range of frequencies studied for each type of normal mode. To illustrate the linear correlation between G_{vib} and frequency, calculated values for G_{vib} are shown in Supplementary Figure 9 (a-d) centered at the following frequencies: 50, 100, 500, and 2000 cm^{-1}. All plotted frequencies range from -50% to 100% of the frequencies listed above and were determined at the following temperatures: 50, 150, 300, 500, and 1000 K.

If we apply a first order Taylor approximation to the nonlinear term we get the following equation. In this approximation, the frequency is linear around some reference frequency ν_O

$$G_{vib} = \frac{1}{2}h\nu + k_B T\, ln\left(1 - e^{\frac{-h\nu_o}{k_B T}}\right) + h\left(e^{\frac{h\nu_o}{k_B T}} - 1\right)^{-1}(\nu - \nu_o)\,. \qquad 42$$

Supplementary Figure 10 displays the sum of Supplementary Equation 41 and 42 over all normal modes for CO adsorbed on 8 different fcc metals at 400 K. The Taylor series for each mode type was centered around the normal mode frequency for CO adsorbed on Cu(111). As can be seen, the vibrational Gibbs Energy under a first order Taylor series approximation is similar to that calculated using the exact equation. As vibrational Gibbs energy correlates linearly with frequency, and frequency correlates with adsorption energy, vibrational Gibbs energy is correlated with adsorption energy.

Coverage calculations were based on the Langmuir bimolecular adsorption isotherm.[37] The effect of vibrations on coverages is studied for monomolecular adsorption of CO, and competitive adsorption between CO and O_2 where O_2 adsorbs dissociatively. The isotherms dictating equilibrium coverages are shown below. All adsorption occurs at the fcc-hollow sites even though CO will often adsorb at atop sites and O_2 can adsorb associatively as well as dissociatively. This system was studied because atomic oxygen adsorption is important in electrocatalysis, among other reactions, and CO poisoning can be a significant problem at both the anode and the cathode.[38-40] At the anode, a proposed solution is to react CO with O_2.[41] The bimolecular competitive Langmuir adsorption isotherm for CO and O_2, valid at low CO and O_2 pressures, where O_2 adsorbs dissociatively to atomic O is given by Supplementary Equation 43[42, 43]

$$\theta_{CO} = \frac{K_{CO}P_{CO}}{1 + K_{CO}P_{CO} + \sqrt{K_O P_{O_2}}}. \qquad 43$$

The equilibrium constants (K_i) were determined from statistical mechanics under the harmonic oscillator and rigid rotator approximations.[36] For the systems studied here, it was assumed that all degrees of freedom of the adsorbed surface species were vibrational. Calculations were done at both 400 K and a pressure of 1 bar. Coverage effects on adsorption energy or frequencies have not been accounted for in this work. The plot in coverage error (Figure 7 of the main text) was based on the scaling relation outlined here, in Supplementary Figure 11.

Supplementary Method – Calculating Frequencies

Frequencies of adsorbed species were calculated using the harmonic approximation and normal mode analysis. Specifically, each adsorbate atom was displaced in Cartesian coordinates by 0.025 Å, the energy of the system was re-calculated, and a mass-weighted Hessian matrix was created.[44, 45] Its eigenvalues, which correspond to the normal mode frequencies, were then computed. As coupling of the motion in Cartesian coordinates generates non-zero off-

diagonals of the Hessian matrix, we developed criteria for assigning one of the normal modes to the perpendicular stretch frequency ($\nu_{\perp}$) of the adsorbate/metal based on the elements of the eigenvector describing the direction of movement. A similar procedure was used to identify parallel frequencies ($\nu_{||}$) and frustrated rotations in the direction perpendicular to the surface ($\delta_{\perp}$), illustrated in Supplementary Figure 12.

The criteria for identifying the perpendicular normal modes are as follows:

1) The direction of displacements in the z-direction is the same for every atom in the adsorbate.
2) The z-displacement magnitude of the adsorbate's atom closest to the metal surface is greater than the z-displacement of any other atom in the adsorbate.
3) The sum of the z-displacements' magnitude is greater than that associated with the x and y directions for the eigenvector describing the frequency given requirements 1 and 2.
4) If the above three criteria were not satisfied for any of the eigenvectors associated with the adsorbates' vibrational normal modes, requirement 2 was relaxed. For frequency mode assignments of CH_2CH_3, criterion 1 was relaxed.

Supplementary References

1. Abild-Pedersen F, Greeley J, Studt F, Rossmeisl J, Munter TR, Moses PG, et al. Scaling properties of adsorption energies for hydrogen-containing molecules on transition-metal surfaces. Phys Rev Lett. 2007;99(1):016105.

2. Ruban A, Hammer B, Stoltze P, Skriver HL, Nørskov JK. Surface electronic structure and reactivity of transition and noble metals. J Mol Catal A: Chem. 1997;115(3):421-9.

3. Jacobsen KW, Norskov JK, Puska MJ. Interatomic interactions in the effective-medium theory. Phys Rev B. 1987;35(14):7423-42.

4. Norskov JK. Effective medium potentials for molecule–surface interactions: H2 on Cu and Ni surfaces. J Chem Phys. 1989;90(12):7461-71.

5. Smith JR, Ying SC, Kohn W. Charge Densities and Binding Energies in Hydrogen Chemisorption. Phys Rev Lett. 1973;30(13):610-3.

6. Nørskov JK, Lang ND. Effective-medium theory of chemical binding: Application to chemisorption. Phys Rev B. 1980;21(6):2131-6.

7. Esbjerg N, Nørskov JK. Dependence of the He-Scattering Potential at Surfaces on the Surface-Electron-Density Profile. Phys Rev Lett. 1980;45(10):807-10.

8. Manzhos S, Jr. TC. Using neural networks to represent potential surfaces as sums of products. J Chem Phys. 2006;125(19):194105.

9. Pradhan E, Brown A. Vibrational energies for HFCO using a neural network sum of exponentials potential energy surface. J Chem Phys. 2016;144(17):174305.

10. Penzar Z, Ekardt W, Rubio A. Temperature effects on the optical absorption of jellium clusters. Phys Rev B. 1990;42(8):5040-5.

11. Rao BK, Jena P. Evolution of the electronic structure and properties of neutral and charged aluminum clusters: A comprehensive analysis. J Chem Phys. 1999;111(5):1890-904.

12. Hammer B, Norskov JK. Theoretical Surface Science and Catalysis - Calculations and Concepts. Advances in Catalysis. 2000;45:71-129.

13. Hammer B, Nørskov JK. Electronic factors determining the reactivity of metal surfaces. Surf Sci. 1995;343(3):211-20.

14. Andersen OK, Jepsen O. Explicit, First-Principles Tight-Binding Theory. Phys Rev Lett. 1984;53(27):2571-4.

15. Jaffé HH, Orchin M. Hybridization in carbon monoxide. Tetrahcdron. 1960;10(3):212-4.

16. Vattuone L, Gambardella P, Burghaus U, Cemič F, Cupolillo A, Valbusa U, et al. Collision induced desorption and dissociation of O[sub 2] chemisorbed on Ag(001). J Chem Phys. 1998;109(6):2490.

17. Backx C, Groot CPMD, Biloen P. Adsorption of Oxygen on Ag(110) Studied by High Resolution ELS and TPD. Surf Sci. 1981;104:300-17.

18. de Mongeot FB, Valbusa U, Rocca M. Oxygen adsorption on Ag(111). Surf Sci. 1995;339:291-6.

19. Baca AG, Klebanoff LE, Schulz MA, Paparazzo E, Shirley DA. Dissociative Adsorption of CO and O2 on Cr(100, Cr(110), and Cr(111) in the Temperature Range 300-1175 K. Surf Sci. 1986;173:215-33.

20. Baddorf AP, Wendelken JF. High coverages of oxygenon Cu(110) investigated with XPS, LEED, and HREELS. Surf Sci. 1991;256:264-71.

21. Sueyoshi T, Sasaki T, Iwasawa Y. Molecular and atomic adsorption states of oxygen on Cu(111) at 100–300 K. Surf Sci. 1996;365(2):310-8.

22. Lu JP, Albert MR, Bernasek SL. The Adsorption of Oxygen on the Fe(100) Surface. Surf Sci. 1989;215:348-62.

23. Colaianni ML, Chen JG, Weinberg WH, Yates J, J.T. Oxygen on mo(110): low-temperature adsorption and high-temperature oxidation. Surf Sci. 1992;279:211-22.

24. Sasaki T, Goto Y, Tero R, Fukui K-i, Iwasawa Y. Oxygen Adsorption states on Mo(1 1 2) surface studied by HREELS. Surf Sci. 2002;502-503:136-43.

25. Stuve EM, Jorgensen SW, Madix RJ. The Adsorption of H2O on Clean and Oxygen-Covered Pd(100): Formation and Reaction of OH Groups. Surf Sci. 1984;146:179-98.

26. Imbihl R, Demuth JE. Adsorption of Oxygen on a Pd(111) Surface Studied by High Resolution Electron Energy Loss Spectroscopy (EELS). Surf Sci. 1985;173:395-410.

27. Gorodetskii VV, Sametova AA, Matveev AV, Tapilin VM. From single crystals to supported nanoparticles in experimental and theoretical studies of H2 oxidation over platinum metals (Pt, Pd): Intermediates, surface waves and spillover. Catal Today. 2009;144(3-4):219-34.

28. Kim CM, Devries BD, Fruhberger B, Chen JG. A HREELS and NEXAFS characterization of the atomic and molecular oxygen species on a vanadium (110) surface. Surf Sci. 1995;327:81-92.

29. Froitzheim H, Ibach H, Lehwald S. Surface vibrations of oxygen on W(100). Phys Rev B. 1976;14(4):1362-9.

30. Wendelken JF. Atomic and molecular states of oxygen chemisorbed on tungsten(112). J Vac Sci Technol A. 1988;6(3):662.

31. Shinn ND, Madey TE. Oxygen Chemisorption on Cr(110). Surf Sci. 1986;176:635-52.

32. Vattuone L, Gambardella P, Valbusa U, Rocca M. HREELS study of O2 molecular chemisorption on Ag(001). Surf Sci. 1997.

33. Sexton BA, Madix RJ. Vibrational Spectra of Molecular and Atomic Oxygen on Ag(110). Chemical Physics letters. 1980;76(2):294-7.

34. Wang X-D, Tysoe WT, Greenler RG, Truszkowska K. A reflection-absorption infrared spectroscopy study of the adsorption of dioxygen species on a silver surface. Surf Sci. 1991;258(1):335-45.

35. Vaska L. Dioxygen-metal complexes: toward a unified view. Acc Chem Res. 1976;9(5):175-83.

36. McQuarrie DA. Statistical Mechanics. Sausalito, CA: University Science Books; 2000.

37. Chorkendorff I, Niemantsverdriet JW. Concepts of Modern Catalysis and Kinetics: Wiley; 2006.

38. Saarinen V, Himanen O, Kallio T, Sundholm G, Kontturi K. Current distribution measurements with a free-breathing direct methanol fuel cell using PVDF-g-PSSA and Nafion® 117 membranes. J Power Sources. 2007;163(2):768-76.

39. Wagner N, Schulze M. Change of electrochemical impedance spectra during CO poisoning of the Pt and Pt–Ru anodes in a membrane fuel cell (PEFC). Electrochimica Acta. 2003;48(25–26):3899-907.

40. Oetjen JF, Schmidt VM, Stimming U, Trila F. Performance Data of a Proton Exchange Membrane Fuel Cell Using H_2/CO as a Fuel Gas. Journal of the Electrochemical Society. 1996;143(12).

41. Baschuk JJ, Li X. Carbon monoxide poisoning of proton exchange membrane fuel cells. International Journal of Energy Research. 2001;25(8):695-713.

42. Fowler RH. A Statistical Derivation of Langmuir's Adsorption Isotherm. Mathematical Proceedings of the Cambridge Philosophical Society. 1935;31(2):260-4.

43. Schwartz SB, Schmidt LD, Fisher GB. CO + O_2 Reaction on Rh(111): Steady-State Rates and Adsorbate Coverages. The Journal of Physical Chemistry. 1986;90(23):6194-200.

44. Steele D. Theory of vibrational spectroscopy. Philadelphia, PA: W. B. Saunders; 1971.

45. Califano S. Vibrational states. New York, NY: Wiley; 1976.
