# Supplementary material for: Scaling relationships and theory for vibrational frequencies of adsorbates on transition metal surfaces
Source: Nat Commun. 2017 Nov 29;8:1842. doi: 10.1038/s41467-017-01983-6 (PMC5705602; doi:10.1038/s41467-017-01983-6)
Supplement: Supplementary file 2 — Description of Additional Supplementary Files [file 41467_2017_1983_MOESM2_ESM.pdf]

Description of Additional Supplementary Files

File Name: Supplementary Dataset 1
Description: The supplementary dataset contains all relevant frequencies and adsorption energies used in generating the figures in the main article and supplementary information. The supplementary dataset includes frequency and energy data for O₂, OHx, NHx, CHx, and CH₂CH₃ species on the (111), (100), and (110) planes of Ag, Au, Cu, Ir, Pd, Pt, Ni and Rh.
